# Mechanism of Dual-Site Recognition in a Classic DNA Aptamer

**DOI:** 10.1021/acs.jcim.4c01389

**Published:** 2024-09-27

**Authors:** Yun-Peng Wang, Leif A. Eriksson, Ru-Bo Zhang

**Affiliations:** † School of Chemistry and Chemical Engineering, Beijing Institute of Technology, South Street No. 5, Zhongguancun, Haidian District, Beijing 100081, China; ‡ Department of Chemistry and Molecular Biology, University of Gothenburg, Medicinaregatan 7b, Göteborg 405 30, Sweden

## Abstract

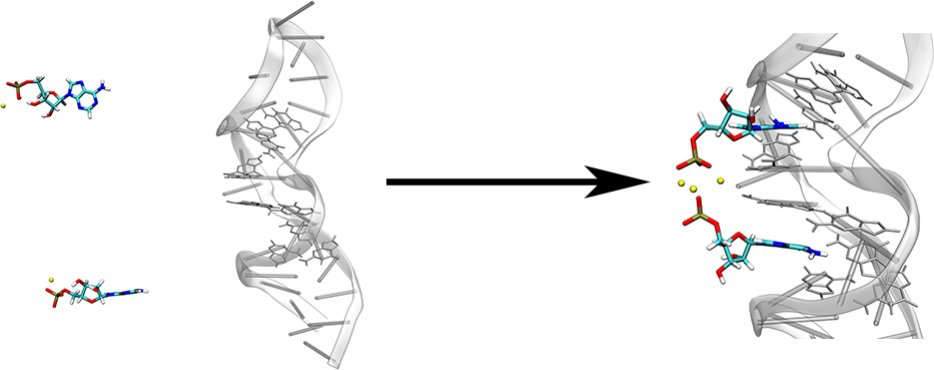

Nucleic acid aptamers possess unique advantages in specific recognition. However, the lack of in-depth investigation into their dynamic recognition mechanisms has restricted their rational design and potential applications in fields such as biosensing and targeted therapy. We herein utilized enhanced sampling molecular dynamics to address affinities of adenosine monophosphate (AMP) to the dual binding sites in the DNA aptamer, focusing on the dynamic recognition mechanism and pathways. The present results indicate that in addition to the widely known intermolecular interactions, inequivalence of chemical environments of the two binding sites leads to slightly higher stability of AMP binding to the site proximal to the aptamer terminus. In the presence of two AMPs captured by the two sites, each binding free energy is enhanced. In particular, an additional hydrogen bond of AMP to A10 is introduced in the dual-site binding complex, which increases the binding energy from −4.25 ± 0.47 to −9.48 ± 0.33 kcal mol^–1^ in the site close to the loop. For the dual-site recognition process, the free energy landscape and minimum free energy pathway calculations elucidate the crucial role of electrostatic interactions between the AMP phosphate groups and Na^+^ ions in positively cooperative binding mechanisms.

## Introduction

Nucleic acid aptamers, comprising highly specific and affinity-bound RNA or single-stranded DNA molecules toward target molecules, have been extensively utilized in the development of biological tools, including biosensors^1^ and targeted therapeutics.^2^ The Systematic Evolution of Ligands by Exponential Enrichment (SELEX) technique^3^ enables the selection of aptamers with specified target affinity from large pools of random nucleic acid libraries through *in vitro* or *in vivo* screening. However, the screening methods face several challenges such as prolonged screening cycles, numerous screening rounds, high error rates, and elevated costs.^4^ In addition, due to the inherent dynamics of nucleic acids, their spatial structures are susceptible to environmental influences, resulting in that aptamers obtained through extensive screening exhibit difficulty in exerting biological functions *in vivo*. Furthermore, within biological organisms, the endogenous nucleic acid aptamers exist primarily in the form of riboswitches present in bacterial genomes.^5^ Riboswitches comprise two components: the nucleic acid aptamer and the expression platform. Upon recognition of signal molecules, the nucleic acid aptamer undergoes conformational changes, thereby modulating the transcription or translation activity of the expression platform, consequently regulating gene expression within the organisms.^6−9^ The chemical essence of aptamers lies in their nucleic acid composition, which can be synthesized from nucleotide polymers, ensuring their inherent programmability.^10,11^ This attribute represents one of the main reasons for the considerable interest in nucleic acid aptamers. In recent years, many studies have focused on chemically modifying aptamers obtained through screening, to enhance their affinity, stability, and other properties.^12−14^

**Scheme 1:**
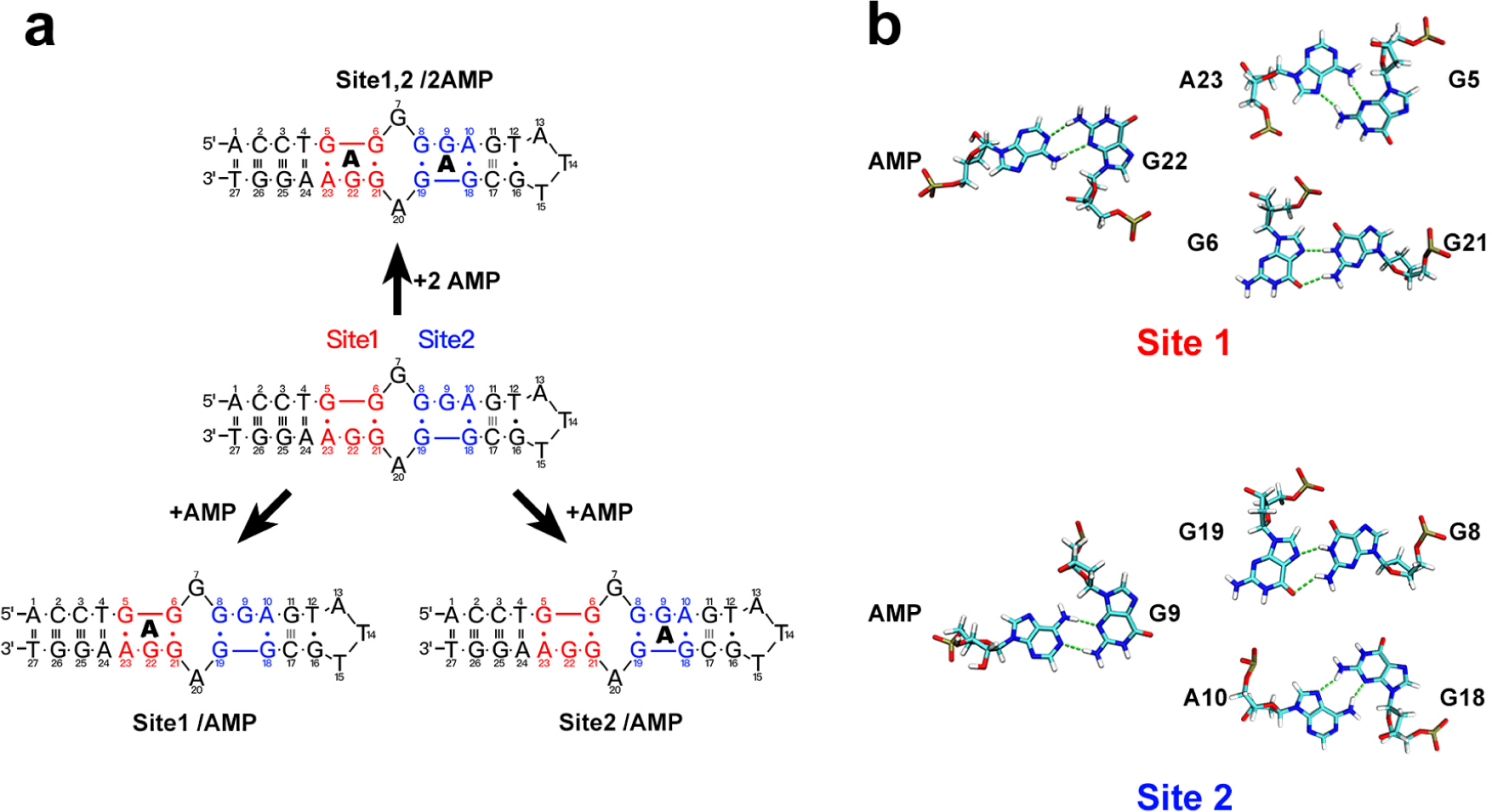
(**a**)Sequence Information of the AMP-DNA Aptamer; The Aptamer Contains the Two Recognition Sites 1 and 2, Represented in Red and Blue, Respectively; (**b**) Non-Canonical Base Pairing at the Recognition Sites

The AMP-DNA aptamer is a DNA nucleic acid aptamer capable of selectively binding adenosine (ADN) and its derivatives (such as AMP, ADP and ATP). It serves not only as the foundation for many biosensors detecting adenosine and its derivatives within organisms but also as an important model for studying the mechanism of nucleic acid aptamers recognizing small molecules. The AMP-DNA nucleic acid aptamer was obtained through *in vitro* screening by Huizenga and Szostak^15^ in 1995, targeting AMP as the molecule of interest. The spatial structure of the aptamer complexed with AMP was determined in 1997 using Nuclear Magnetic Resonance (NMR) methods,^16^ which provided crucial insights into the recognition pattern of the aptamer toward AMP. NMR structures indicated that the aptamer comprises two AMP recognition sites. Each site is primarily composed of two G and one A, along with two additional G for stabilizing the recognition site, as illustrated in Scheme 1. Taking Site 1 as an example, AMP forms a noncanonical base pair with the G22 base of the recognition site, stabilized by π–π stacking interactions with the G6 and A23 bases. In addition, the G6 and G21 bases form a reverse Hoogsteen G·G pair, and the G5 and A23 bases form a sheared G·A mismatch, respectively, creating a stable binding pocket for the specific recognition of adenosine derivatives. The notation Site 1 (or Site 2)/AMP and Sites 1,2/2AMP are herein used to denote the single-site and dual-site recognition models, respectively.

One of the significant characteristics of this DNA aptamer is its ability to bind to two ligands simultaneously, known as dual-site recognition. Experimental research on the dual-site recognition capability of nucleic acid aptamers is currently limited, possibly due to the difficulty in distinguishing ligand-binding sites and controlling them using conventional experimental techniques. Existing studies primarily rely on base mutations and isothermal titration calorimetry (ITC) to compare and analyze the thermodynamic parameters of wild-type DNA aptamers and mutant aptamers binding ligands at two sites. Liu et al.^17^ achieved single-site recognition capability by rationally designing DNA aptamers, wherein they selectively removed one of the binding pockets from the aptamer structure using classic Watson–Crick base pairing. According to the thermodynamic parameters obtained from ITC measurements, the single-site mutant and wild-type DNA aptamers exhibited comparable affinity for adenosine ligands, approximately −6.5 kcal mol^–1^, suggesting that the two recognition pockets possess highly similar and independent recognition capabilities without any synergistic effects. On the other hand, Slavkovic et al.^18^ replaced guanosine, which can form hydrogen bonds with adenosine ligands at the recognition site, with inosine, while constructing single-site recognition aptamers, thereby preserving some degree of disorder in the other recognition site. The affinity of the inosine-replaced single-site recognition aptamers varied, with dissociation constants of 188 ± 37 μM (Site 1) and 212 ± 69 μM (Site 2), which was interpreted as a difference in the recognition capabilities of the two sites. However, taking the error bars into consideration, these values are so close that a difference in affinities of the two sites toward AMP cannot unambiguously be concluded. More importantly, the obtained ITC data^18^ fit a cooperative binding mode with a Hill coefficient of 1.3. Consequently, a positive cooperative binding mechanism following a layout shifting binding model was proposed, wherein the binding of the first ligand facilitates tighter binding of the second ligand. Controversies surround the mechanism of dual-site recognition can be attributed to the presence of multiple conformations of the wild-type aptamer. Due to the high flexibility brought out by the very short persistence lengths of single-stranded oligonucleotides, and influence of the dynamic nature of the local structures of the DNA aptamer such as fluctuations of the loop and bulge regions, studying the mechanisms by which they recognize small molecules poses several challenges. Mutational and modification studies of nucleic acid aptamers may alter their inherent complex spatial structures. Moreover, environmental effects must also be considered for the successful use of the aptamer in the recognition of small molecules, aspects which have essentially been ignored.

In this study, we have focused on the wild-type 27-nt DNA aptamer-AMP_2_ complex to construct a dual-site binding model. To construct single-site recognition models, we removed the AMP ligands from either of the two recognition sites, respectively. By employing all-atom molecular dynamics simulations combined with well-tempered metadynamics with extended adaptive biasing force (WTM-eABF) enhanced sampling, we investigated the single- and dual- site recognition processes of the nucleic acid aptamer, finding that the two sites are almost equivalent, with Site 1/AMP slightly more stable than Site 2/AMP. The small difference is related to the inequivalence of the chemical environments around the two identical Sites. Interestingly, the stability of Sites 1,2/2AMP is enhanced by additional interaction between the phosphate-sugar fragments of both AMP’s and the residues in the sites, explaining why the second molecule is more facile to be recognized for Site 1(2)/AMP. A weak positive cooperative binding mechanism for the dual-site recognition is proposed based on bridging phosphate anion—sodium cation—phosphate anion structures observed in this study. Through comparative analyses, the crucial role of the phosphate of the AMP molecules in the weak positive cooperative binding mechanism is further corroborated.

## Computational Methods and Details

As initial structures of our molecular dynamics simulations for the dual-site recognition model we used the data from NMR experiments by Lin and Patei^16^ (PDB ID: 1AW4). The single-site recognition models were generated by removing the AMP ligands from sites 1 or 2, respectively. The complex of the 27-nucleotide aptamer with AMP molecule(s) was placed in a cubic box measuring 90 Å × 90 Å × 90 Å, containing approximately 14 000 TIP3P water molecules. To simulate the ionic strength of biological environments, we added Na^+^ and Cl^–^ ions to represent a 0.15 M NaCl solution, after first neutralizing the system’s internal charges with Na^+^ ions.

**Figure 1:**
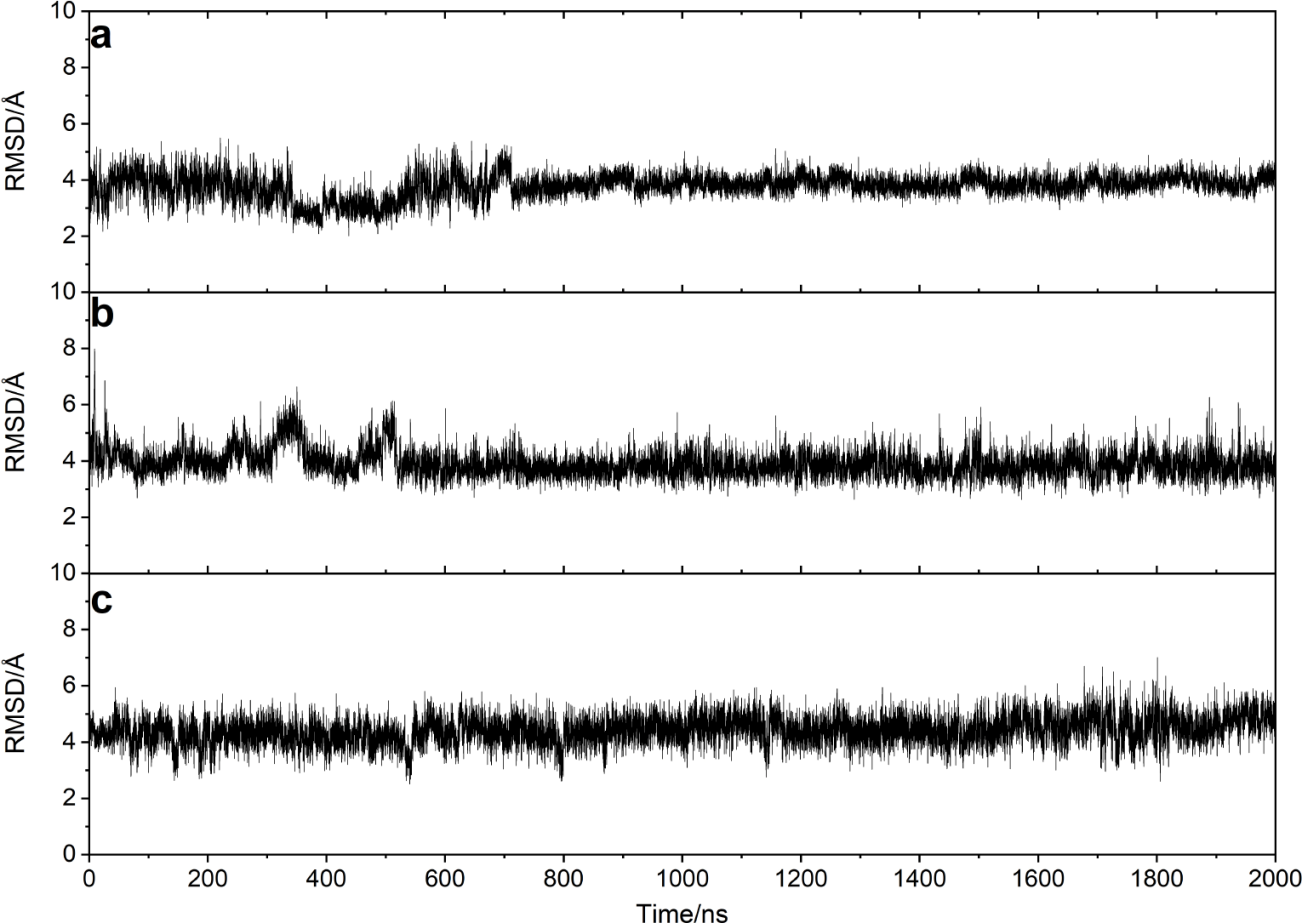
RMSDs of the aptamer-AMP complexes relative to the initial structures over the course of 2 μs equilibrium dynamics simulations. (a) Dual-site recognition model with AMP simultaneously bound to Sites 1 and 2. (b) Single-site recognition model with AMP bound to Site 1. (c) Single-site recognition model with AMP bound to Site 2.

The systems were preprocessed and equilibrium simulations conducted using the following procedure. Initially, the systems underwent energy minimization with 5000 steps of steepest descent. Subsequently, in the NVT ensemble, we restrained the solute while heating the solvent to 300 K over 100 ps. Then, in the NPT ensemble at 300 K and 1 atm, we gradually relaxed the harmonic restraints on the solute with force constants of 10, 5, 1, 0.1, and 0 kcal mol^–1^ Å^–2^, facilitating controlled solute release. Each relaxation process lasted 500 ps. Following this, each system underwent long equilibration simulations (2 μs), during which the stability of the complex conformations in the dynamic trajectories was characterized relative to the initial structures of the aptamer-AMP complex using Root Mean Square Deviation (RMSD) analyses, seen in Figure 1. This process was repeated four times, with each replicate yielding similar simulation results. RMSD plots of the replicates are shown in Figures S1–S3. For the dual-site recognition model, the average of the four RMSD curves varies around 3.6 Å. The RMSD fluctuations around 1650 ns in replica a, 900 and 1800 ns in replica b, and 500 and 700 ns in replica d (Figure S1) are mainly caused by the dissociation/association of the terminal A1–T27 and C2–G26 base pairs. Part of the reasons for the RMSD fluctuation of the four replicas also lies in the fact that the G7 and A20 bases are free to flip inside and outside the aptamer under the influence of solvents, leading to small variations in the RMSD. The corresponding Root Mean Square Fluctuations (RMSF) for each nucleotide, seen in Figure S4, also account for the variation in RMSDs across the four replicas. In the system Site1/AMP, the RMSD of the whole aptamer is slightly increased. Since Site 2 is not occupied, this results in increased fluctuations of G7, as shown in Figure S5, resulting in the mean of the RMSD of the four replicates varying around 4.1 Å. A corresponding situation is seen for the empty site in the Site 2/AMP system, and RMSD values vary around 4.4 Å, (Figures S3 and S6). In addition, looking at the RMSD curves of only the recognition sites, shown in Figures S7–S9, we note that the nucleotide fluctuations in the loop, bulge and end regions of the aptamer actually have relatively small effects on the structural changes at the recognition site. These are very helpful for an effective analysis of the interaction between AMP and the aptamer. The total duration of the equilibrium dynamics simulations was 24 μs.

**Scheme 2:**
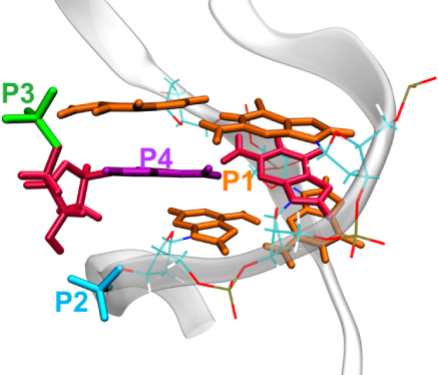
Approaching Angle is a Pseudo Dihedral Angle Defined Using Four Centers of Mass (P1, P2, P3, and P4) In this context, P1 represents the center of mass of the base parts of nucleotides G6, G21, G5, and A23. P2 is the center of mass of a group comprising the phosphate atom (P) in the backbone of A24 and its four bonded oxygen atoms. P3 denotes the center of mass of the phosphate group of AMP, while P4 represents the center of mass of the base of AMP. This angle provides a quantitative measure of the spatial orientation when AMP approaches site 1.

In order to understand the dynamic process of aptamer recognition of AMP, we employed the WTM-eABF enhanced sampling algorithm for free energy calculations. This algorithm is based on the extended adaptive biasing force (eABF) and metadynamics methods, which have been successfully applied to various systems,^19−26^ including nucleic acids. The choice of collective variables is crucial for the WTM-eABF enhanced sampling algorithm. For the one-dimensional Potential of Mean Force (PMF) calculations, we used the distances between the centroid of the AMP base and the centroid of the base of G22 (Site 1) or G9 (Site 2), respectively, as the collective variables. For the two-dimensional free energy landscape calculations studying the recognition pathways, we used the aforementioned centroid distance along with a pseudodihedral angle to describe the approaching angle, as shown in Scheme 2. For the two-dimensional free energy landscape calculations studying the recognition mechanism, we used as collective variable on the *x*-axis the distance between the centroid of the first AMP base and the centroid of the base of G22 (Site 1), while the *y*-axis collective variable was the distance between the centroid of the second AMP base and the centroid of the base of G9 (Site 2). For the one-dimensional PMF calculations for single-site recognition and the two-dimensional free energy landscape calculations for single- and dual-site recognition pathways, each enhanced sampling simulation lasted 300 ns and was repeated in triplicate. The convergence test plots (Figures S10 and S11) display PMF curves at various simulation times. The system samples repeatedly along a predefined collective variable within its specified range. Initially, due to insufficient sampling, the PMF curves differ significantly. After 300 ns, the collective variable covers the entire sampling range, and the PMF curves show minimal changes over time (deviations within 1 kcal/mol among three 300 ns replicas). We consider these simulations to be converged and thus used 300 ns as the sampling time for each replica.

For the two-dimensional recognition process free energy landscape calculations of AMPs in the dual-site recognition pathways, each enhanced sampling simulation lasted 1200 ns. The total duration of the enhanced sampling simulations reached 7.8 μs. Details of the simulation parameters are provided in the supporting material.

In the molecular dynamics simulations described above, we utilized the Amber OL15 force field^27,28^ modified for DNA, and the Amber GAFF force field^29,30^ for AMP. Initial topology files were generated online using the CHARMM-GUI tool.^31,32^ The ligand structures were optimized using Gaussian16 at the B3LYP-D3(BJ)/def2-TZVP level^33−35^ and RESP2 charges were computed using the Multiwfn program.^36^

Long-range electrostatic interactions were treated with the Particle Mesh Ewald (PME) algorithm^37^ with a van der Waals cutoff radius of 14 Å. Bond lengths involving hydrogen atoms were constrained using the LINCS algorithm.^38^ The Berendsen^39^ and V-rescale^40^ algorithms were employed to control pressure and temperature at 1 atm and 300 K, respectively.^41^

The simulations were performed with a fixed time step of 2 fs using Gromacs 2021.4^42−44^ with the Colvars^45,46^ module patched and accelerated with GPU. Trajectory visualization and analysis were carried out using VMD 1.9.3.^45,47^ The minimum free energy pathways were explored using the MULE program.^22^

## Results and Discussion

### Effects Brought Out by Inequivalence of the Chemical Environments Around Recognition Sites

The two recognition sites in the aptamer exhibit symmetry in terms of nucleic acid sequence and secondary structure. One recognition site, defined as Site 1 seen in Scheme 1, is proximal to the 5′ and 3′ ends, while the other (Site 2) is near the loop. The structural differences in spatial arrangement may imply distinctions between the two recognition sites. In addition, their linkers, G7 and A20, are highly flexible, which is also a characteristic of the aptamer. The region connected by Site 1 and pointing to the 3′ and 5′ ends is more rigid than that connected by Site 2 and pointing to the loop, due to the fact that the former is composed of Watson–Crick base pairs, whereas the latter is composed of a higher degree non-WC base pairs, seen in Scheme 1. This inequivalence of the chemical environments of the recognition sites could lead to difference of affinities of the two sites to AMP. To this end, we first computed the PMF curves separately to estimate the binding free energies of the single-site recognition processes.

**Figure 2:**
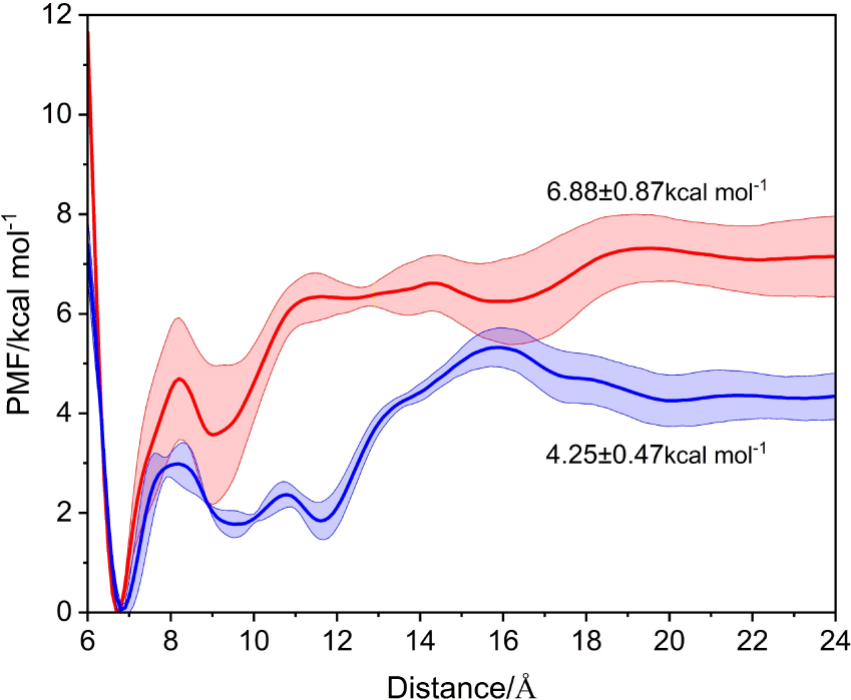
Potential of Mean Force (PMF) curves of the single-site recognition models. The red curve represents the variation of the system’s free energy with the distance between AMP and the centroid of base G22 when one AMP ligand binds to Site 1, and the blue curve correspondingly between AMP and the centroid of base G9 when one AMP ligand binds to Site 2. The error bars in the PMF curves are calculated based on the standard deviation from three repeated PMF calculations.

As seen in Figure 2, the PMF curves reveal that the binding free energy of AMP at Site 1 is −6.88 ± 0.87 kcal mol^–1^, whereas at Site 2, it is −4.25 ± 0.47 kcal mol^–1^. This indicates a slightly higher stability of AMP in binding at Site 1, consistent with the trend observed in the measured dissociation constants, being 188 ± 37 μM for Site 1 and 212 ± 69 μM for Site 2, respectively,^18^ and can be understood in terms of the inequivalence of the chemical environments of the recognition sites. Using Watson–Crick base pairs to extend the length of the nucleic acid strands at both ends of the recognition sites, the affinities of the two sites to AMP should converge, as shown in Liu et al.^17^ The present average binding free energy at both sites is −5.6 kcal mol^–1^, showing only a minor deviation from the binding free energy −6.5 kcal/mol measured using isothermal titration calorimetry (ITC),^17,48^ and consistent with the binding free energy −5.4 kcal/mol calculated in our previous study.^26^ This consistency adds reliability to the binding free energy calculations with the WTM-eABF enhanced sampling algorithm used herein. The convergence test results are shown in Figures S10 and S11.

**Figure 3:**
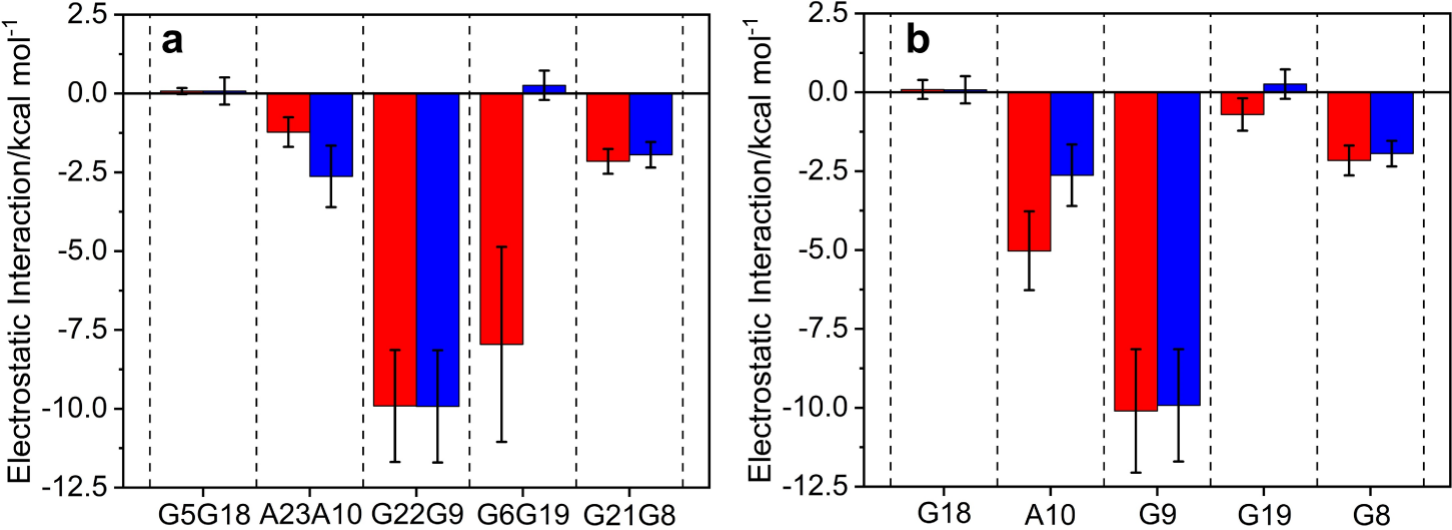
Decomposition of electrostatic interactions between AMP and (a) Residues in Site 1 (in red) or 2 (in blue) in the single-site recognition model, or (b) Residues of Site 2 in the Site 2/AMP (in blue) and in the Sites 1,2/2AMP (in red).

**Figure 4:**
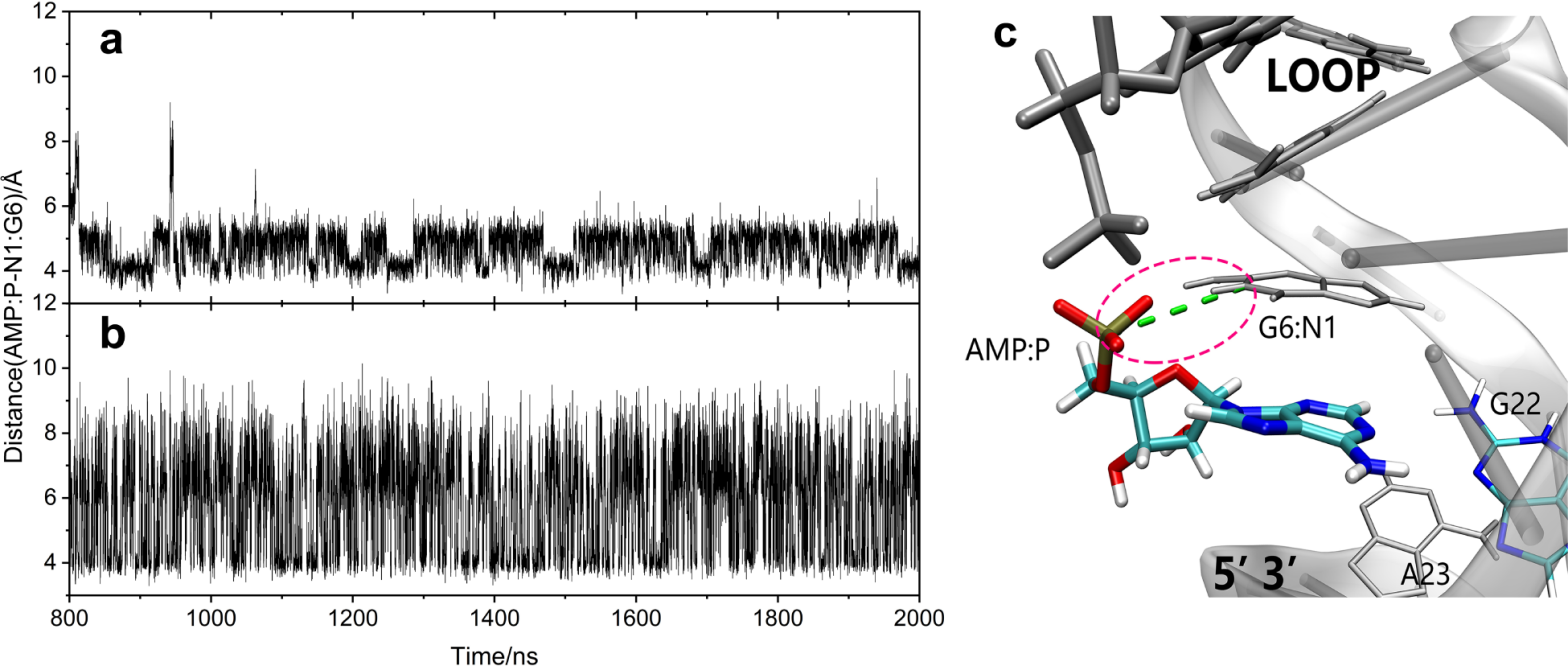
Distance between the phosphorus atom of AMP in Site 1/AMP and G6N1 is (a) 4.71 ± 0.50 Å in the dual-site recognition model and (b) 5.79 ± 1.37 Å in the single-site recognition model, with statistical analysis conducted after the overall RMSD stabilized at *t* = 800 ns; (c) the interaction between AMP and G6N1 in the dual-site recognition model.

To explore the influence brought out by the inequivalence of the chemical environments of the two recognition sites, we conducted interaction energy decomposition analysis based on the equilibrium trajectories of the single-site models (Table 1). Although interrelations between potential energies and free energies are not straightforward, the aim here is to explore the contribution of the interaction potential to the interaction free energy. The van der Waals (VdW) interactions between AMP and residues within a 4 Å radius at Site 1 were calculated to be −14.22 ± 2.31 kcal mol^–1^, and at Site 2–14.90 ± 2.17 kcal mol^–1^. Thus, there is no significant difference in van der Waals interactions between the two recognition sites, implying that the key bases at the two recognition sites in the aptamer should maintain their chemical environment and structural integrity, particularly in terms of π–π interactions, during AMP recognition at either site. However, in contrast, the electrostatic (Elec) interactions between AMP and residues within a 4 Å radius were −21.16 ± 10.06 kcal mol^–1^ at Site 1, whereas at Site 2 they were −14.22 ± 6.15 kcal mol^–1^, indicating that the slightly stronger affinity of Site 1 over Site 2 is due to a disparity in electrostatic interactions. According to previous analyses based on structural and theoretical calculations, the electrostatic interaction arise from the hydrogen bonds formed between the adenine base of AMP and G9/G22 in the G/A trans Hoogsteen mismatch.^26^ Decomposition of the electrostatic interactions between AMP and its surrounding residues presented in Figure 3a, shows that the electrostatic interaction between AMP and G22 at Site 1 is −9.91 ± 1.77 kcal mol^–1^, and between AMP and G9 at Site 2–9.92 ± 1.78 kcal mol^–1^, implying that the hydrogen bonds between AMP and G22/G9 are not the reason for the observed difference in electrostatic interactions. Further inspection instead revealed that the special interaction of −7.96 ± 3.10 kcal mol^–1^ between AMP and G6 at Site 1, is completely nonexistent between AMP and the corresponding G19 at Site 2. The global structure and the interaction between the two recognition sites and AMP are illustrated in Figure S12. To further explore the underlying reasons for this, we statistically analyzed the distances between AMP and the G6 guanine nucleotide at Site 1 along the equilibrium dynamics trajectories. We observed intermittent electrostatic-dominated interaction between the oxygen anion of the phosphate group in AMP and N1H of the G6 base, as depicted in Figure 4. Note that the interaction fluctuates along the trajectories because of the solvent exposure of the AMP phosphate group, thus affecting the distance 5.79 ± 1.37 Å between the phosphorus in AMP and the G6N1 base. Based on this analysis, it appears that the additional (G6)N1H···O^–^(PO_4_^–^) AMP interaction to a certain extent contributes to the increased stability of the AMP–Site 1 binding.

**Table 1:** Interaction Energies (kcal mol^–1^) between AMP and the Recognition Sites in the Single-Site and Dual-Site Recognition Models

Interaction Energy	1AMP-Site1	1AMP-Site2	2AMP-Site1	2AMP-Site2
vdW	–14.22	–14.90	–14.23	–14.60
	(±2.31)	(±2.17)	(±2.24)	(±2.17)
Elec	–21.16	–14.22	–16.40	–17.88
	(±10.06)	(±6.15)	(±5.68)	(±6.11)
Total	–35.38	–29.12	–30.63	–32.48
	(±10.32)	(±6.52)	(±6.11)	(±6.48)

**Figure 5:**
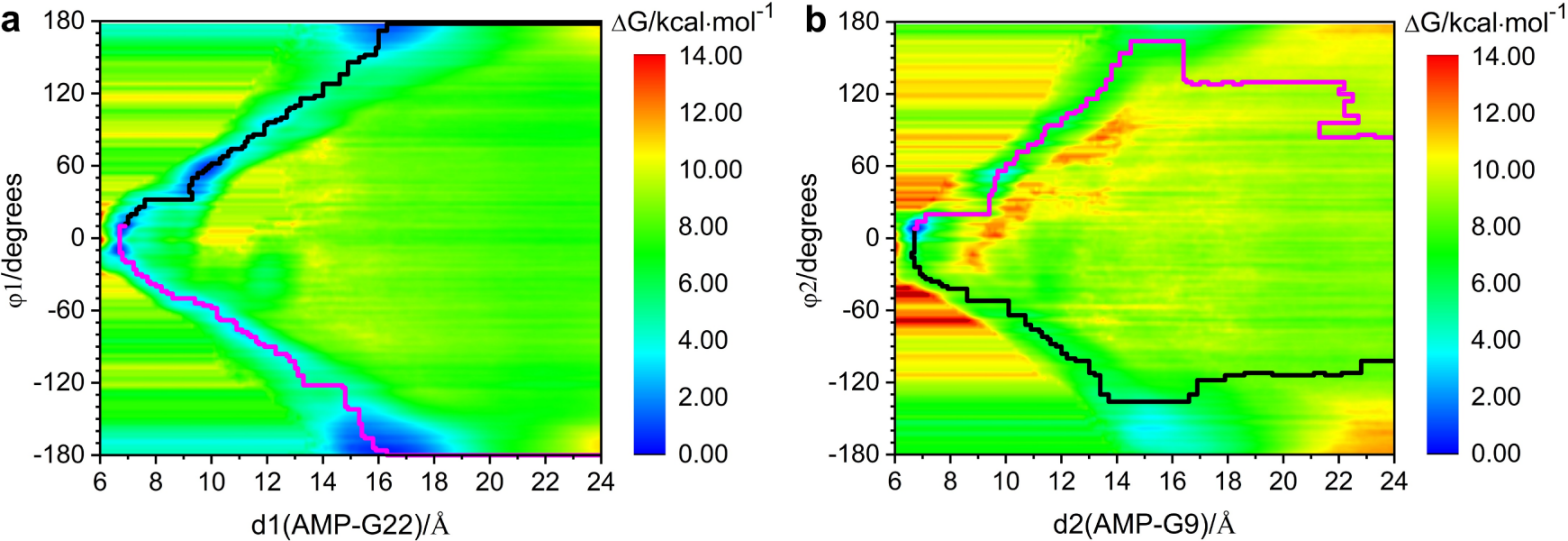
Free energy landscape for (a) Site 1 recognition of AMP in the Site 2/AMP system, and (b) Site 2 recognition of AMP in the Site 1/AMP system. The black curve represents the minimum free energy pathway calculated using the MULE program, while the pink curve indicates the suboptimal free energy pathway.

The structural impact on the complex when Site 1 binds AMP, on the ability of Site 2 to bind the other AMP, and vice versa, is important in order to understand the aptamer structure–function relationship. Based on Figure 5a, the binding free energy of AMP at Site 1 is −8.35 ± 0.60 kcal mol^–1^ when an AMP is already bound to Site 2, compared to −6.31 ± 0.20 kcal mol^–1^ for Site 1 + AMP → Site 1/AMP, indicating an slightly increased stability of 2.04 kcal mol^–1^. Similarly, the binding free energy of AMP to Site 2 is −4.67 ± 0.15 kcal mol^–1^, which is more than doubled to −9.48 ± 0.33 kcal mol^–1^ (Figure 5b) when a molecule of AMP is already bound to Site 1. The rationale for the excessive stability can be seen through a comparative analysis of the equilibrium dynamics trajectories of the single-site and dual-site recognition models. As displayed in Figure 4, there is enhanced interaction between the phosphate group of AMP and G6N1 at Site 1 of the Sites 1,2/2AMP complex, compared to the case in the Site 1/AMP complex. The distance between the phosphorus atom of AMP and G6N1 decreases from 5.79 ± 1.37 Å in the single-site model to 4.71 ± 0.50 Å in the dual-site recognition model and the fluctuation due to solvation is strongly reduced.

**Figure 6:**
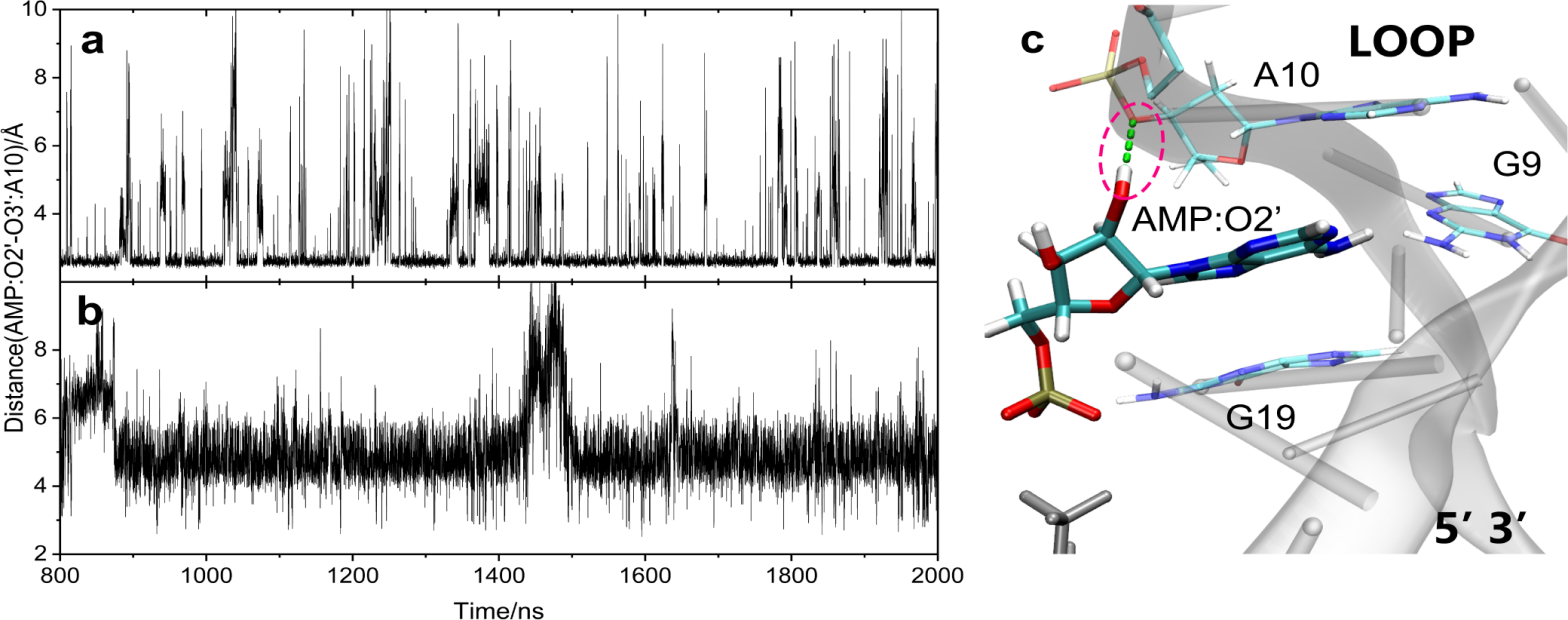
Distance between the O2’ atom of AMP in Site 2 and the O of the phosphate in A10 is (a) 3.05 ± 0.73 Å in dual-site recognition model, and (b) 5.07 ± 0.72 Å in the single-site recognition model (b), with statistical analysis conducted after the overall RMSD stabilized at *t* = 800 ns; (c) interaction between AMP and O of PO_4_^–^ in A10 in the dual-site recognition model.

A comparative residue decomposition analysis of the electrostatic interactions was also conducted between AMP and the residues surrounding Site 2 in the Site 2/AMP and Sites 1,2/2AMP complexes (Figure 3b). The electrostatic interactions between AMP and residues G9, G18, G8 and G19 showed no significant difference between the single and dual-site recognition models. However, there is a notable difference in the electrostatic interaction between AMP and residue A10, which increases from −2.67 kcal mol^–1^ in the Site 2/AMP recognition model to −5.02 kcal mol^–1^ in Sites 1,2/2AMP, attributed to an additional hydrogen bonding between the ribose O2’ of AMP and the phosphate backbone of A10 with a length of 3.05 ± 0.73 Å (Figure 6). Hence, the large electrostatic interaction between the phosphate groups of the two AMP molecules and G6N1 and A10 in the aptamer, respectively, suggests that the local binding of an initial AMP to the aptamer favors the formation of Sites 1,2/2AMP through an increased binding free energy of the second AMP to the vacant Site 1 or 2. Moreover, the impetus for this enhanced recognition of the second AMP can be attributed to intermolecular interactions rather than to π–π interaction between the aptamer and AMP or within the aptamer self.

### Differences in Recognition Pathways

Base flipping is known to influence the stability of double-strand nucleic acid structures. In the current context, flipping pathways correspond to different base orientations in the molecular recognition. Inspired by previous studies,^49−51^ we became intrigued by the possibility of different pathways of the noncovalently bound AMP ligands approaching the DNA recognition sites during the molecular recognition processes. Notably, based on the distance between the AMP-G9/G22 base centroids, the enhanced sampling simulations revealed two different directions of AMP approaching the sites. To elucidate the difference in the recognition pathways toward AMP of each site in the aptamer, we used a two-dimensional collective variable (CV) composed of the center-of-mass distance (d) and the approaching angle (φ). Specifically, d1 (d2) represents the distance between AMP and the G22 (G9) base centroid. The definition of φ1 (φ2) follows the convention established by Song et al.^51^ for the CPDb dihedral angle, describing AMP’s different recognition pathways. CPDb is a modified COM pseudodihedral (CPD) angle definition used as a collective variable to describe the base flipping process in nucleic acids. It is defined by the dihedral angle formed by four centroids: the centroids of two Watson–Crick paired bases adjacent to the flipping base, the centroid of two backbone phosphate groups adjacent to the flipping base, and the base of the flipping nucleotide. When φ ranges from 0 to 180°, AMP is recognized from the solvent side; and when φ ranges from −180° to 0, AMP is recognized from the near-backbone side.

**Figure 7:**
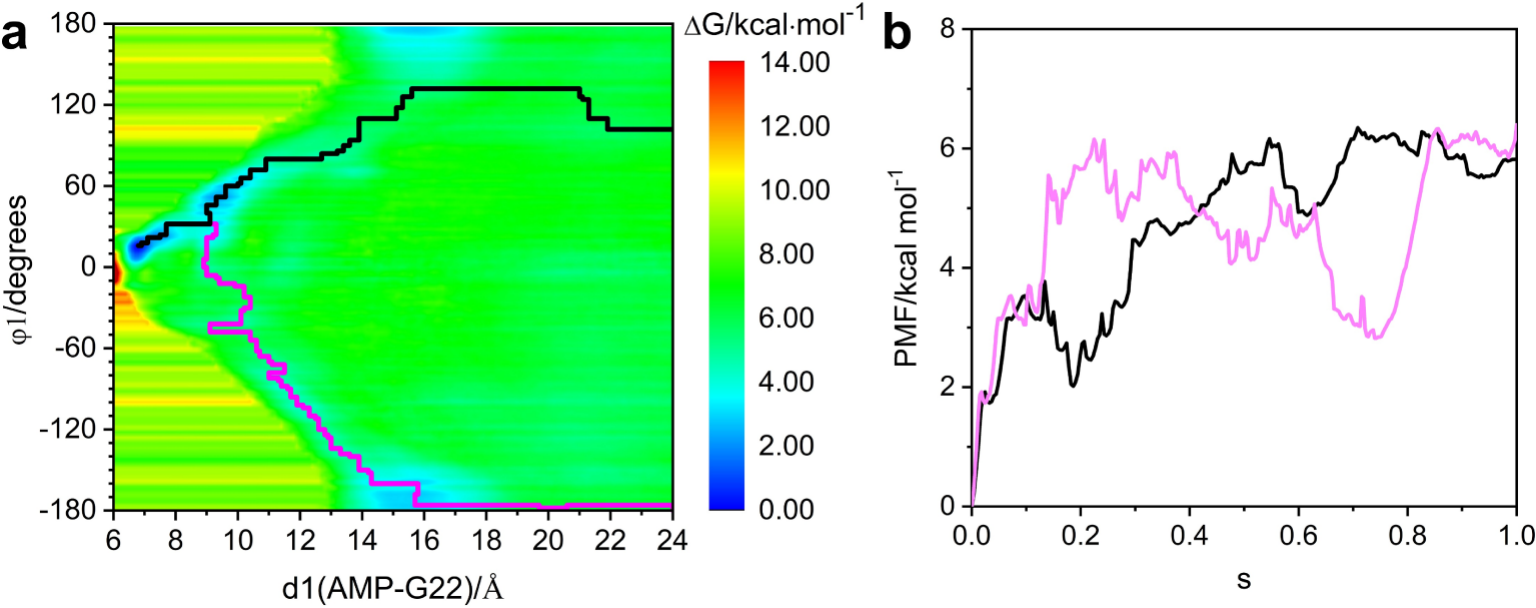
(a) Free energy landscape of Site 1 recognition of AMP in the single-site recognition model. The black curve is the minimum free energy pathway and represents the optimal recognition pathway. The pink curve displays the suboptimal recognition pathway; (b) PMF curves along the minimum free energy pathway (black) and the suboptimal recognition pathway (pink). The horizontal axis ‘s’ represents the progress along the Path Collective Variable (Path CV). Path CV is a method used to describe the progress of a system along a predefined pathway. ‘s’ is a dimensionless parameter used to measure the progress along this path, with ‘s = 1’ corresponding to the initial state and ‘s = 0’ corresponding to the final state.

Using the algorithm developed by Fu et al.,^22^ the minimum free energy pathway (MFEP) was explored, and is displayed in Figure 7. Our results demonstrate that AMP at Site 1 tends to approach from the solvent side (blue region in Figure S13), as shown by the solid black line in Figure 7. For comparison, we also computed the energy changes of the suboptimal recognition pathway related to AMP approaching at the near-backbone side and is shown by the solid pink curve in Figure 7. The data reveals that recognition from the solvent side entails a smaller energy barrier, ca. 1.2 kcal mol^–1^, indicating a clear kinetic advantage. The binding free energy obtained for the AMP ligand at Site 1 is −6.31 ± 0.20 kcal mol^–1^, consistent with the −6.88 ± 0.87 kcal mol^–1^ obtained from one-dimensional CV-enhanced sampling based on the distance between centroids (Figure 2). The same method was employed for the study of the recognition pathway of the AMP ligand at Site 2. As presented in Figure S14, the free energy landscape and the PMF curve of the minimum free energy pathway show that in the single-site recognition model, the AMP ligand also tends to approach to Site 2 from the solvent side (blue region in Figure S15), with a binding free energy of −4.67 ± 0.15 kcal mol^–1^. This result is again in agreement with the above calculation of the free energy −4.25 ± 0.47 kcal mol^–1^ (Figure 2). The present results furthermore indicate that the pathways of AMP binding recognized by Site 1 or Site 2 tend to occur with the nucleobase oriented toward the solvent side, consistent with our previous study.^26^ AMP oriented toward the solvent side makes it more favorable for water molecules to migrate into/out of the aptamer pocket, whereas approaching the near-backbone side may lead to interactions between the adenine of AMP and the phosphate backbone, hindering further binding into the recognition pocket.

The same method was also applied to explore the optimal recognition pathways of AMP to Site 1 or Site 2 to form the dual Sites 1,2/2AMP. Their free energy landscapes are displayed in Figure 5 and recognition pathways are displayed in Figures S16 and 8, respectively. Interestingly, AMP approaching Site 1 still tends to flip toward the solvent side, whereas AMP binding to Site 2 now instead tends to approach the near-backbone side. Based on the simulation trajectories, the reason inferred for this is that in the Sites 1,2/2AMP model, A20 near Site 2 occupies the solvent side of the recognition pathway and forms an A20:N6···N3:G19 hydrogen bond, thereby increasing the steric hindrance energy barrier for AMP approaching the solvent side by approximately 1.5 kcal mol^–1^ (Figure 5b). The current results of the recognition pathway provide the basis for the dual site recognition mechanism of AMP.

**Figure 8:**
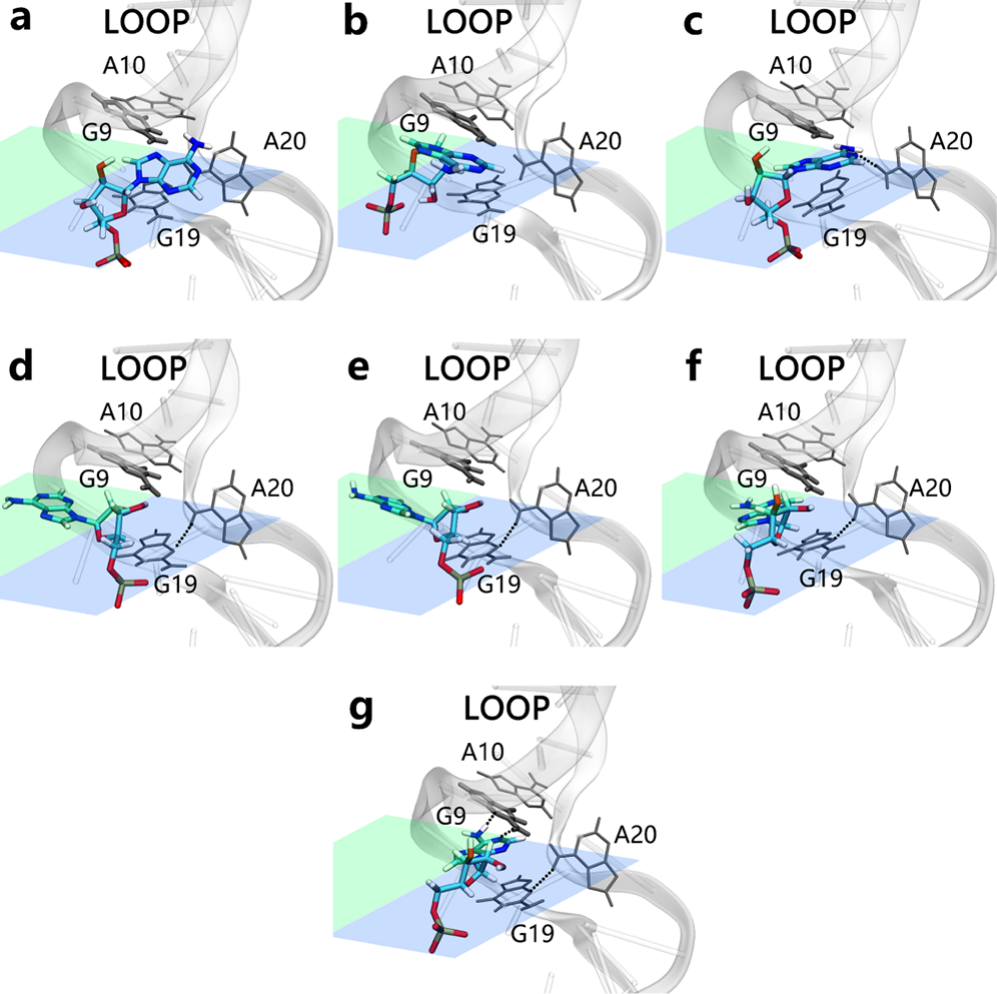
Different pathways of AMP molecule recognition at Site 2 in the dual-site recognition model. The upper region (a, b, c) is represented in blue to indicate the pathway of ligand binding from the solvent side, φ ranging from 0 to 180°, which corresponds to a suboptimal recognition pathway that requires the system to overcome additional energy barriers due to the hydrogen bond A20:N6···N3:G19. The lower region (d, e, f) is marked in green to signify the pathway of ligand binding from the near-backbone side, φ ranging from −180°to 0, corresponding to the minimum free energy pathway. Panel (g) at the bottom represents the final stable binding state.

### Recognition Mechanism in the Dual-Site Model

**Figure 9:**
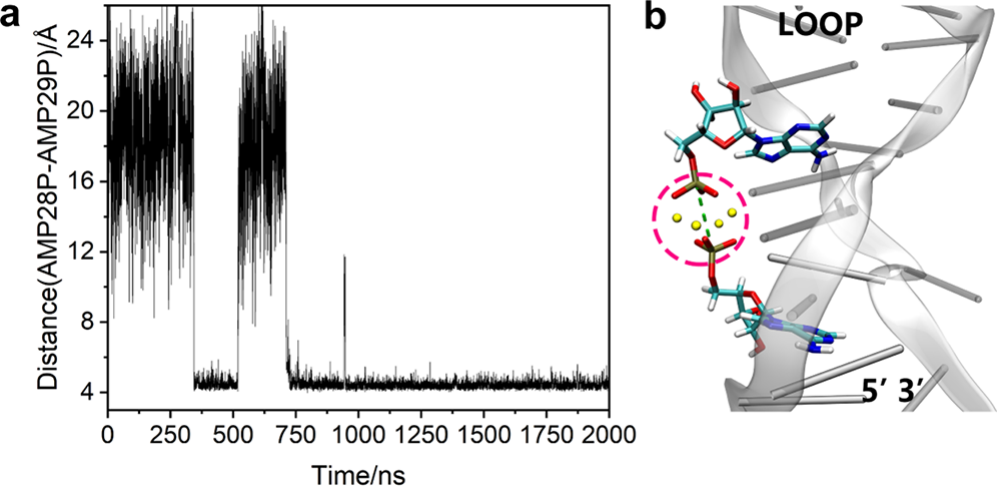
(a) Distance between the P atoms of the two AMP ligands during the 2 μs simulation in the dual-site recognition model; (b) dual-site model showing the measured distance between the AMP phosphates (green dashed line) and the interacting Na^+^ ions (yellow spheres).

Na^+^ ions were found to significantly mediate interaction between the phosphate groups of the two AMP molecules in the Sites 1,2/2AMP model. The distance between the phosphate groups of the two AMP molecules was recorded during the molecular dynamics trajectories of the dual-site recognition model, shown in Figure 9. After initial rapidly fluctuating orientations, a long-lived stable complex is formed from 800 to 2000 ns, with the distance between the P atoms being 4.45 ± 0.37 Å. This stabilizing interaction appears highly relevant for the dynamic mechanism of dual-site recognition by the aptamer.

**Figure 10:**
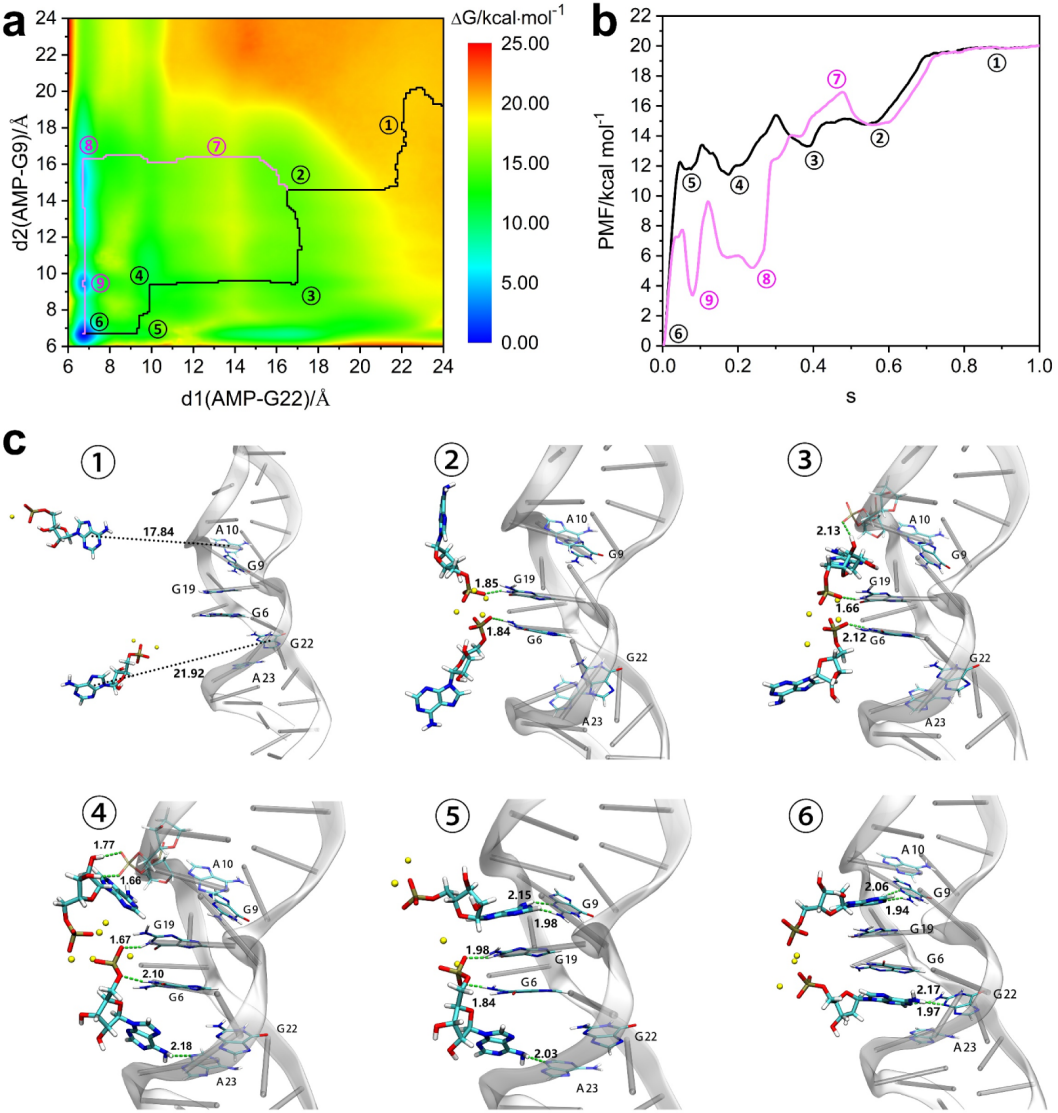
(a) Free energy landscape of the dual-site recognition mechanism. The horizontal axis represents the distance between one AMP and the centroid of base G22 in Site 1 and the vertical axis the distance between the second AMP and the centroid of base G9 in Site 2. The black curve represents the minimum free energy pathway, and the pink a suboptimal recognition pathway; (b) PMF curves along the minimum free energy pathway (black) and the suboptimal recognition pathway (pink). The horizontal axis represents the variable ‘s’ indicating the progress along the CV path. (c) Snapshots of the cooperative recognition process in the dual-site recognition model. The snapshots are arranged according to the order of ligand binding, corresponding to steps ①, ②, ③, ④, ⑤, and ⑥ in (b) along the minimum free energy pathway, respectively.

Using the WTM-eABF enhanced sampling algorithm, biases to both AMP molecules at the two recognition sites were simultaneously applied to simulate the process of the aptamer simultaneously recognizing two AMP ligands to Sites 1 and 2. Based on the free energy landscape as presented in Figure 10a, we computed the minimum free energy pathway and the PMF along these pathways, presented in Figure 10b. Relevant structures along these pathways are displayed in Figure 10c. The minimum free energy pathway (solid black curve) reveals that during the dual-site recognition process, the ligands initially move freely in the solution and approach the aptamer. As the phosphate groups of the two AMP ligands come closer to each other, bridging sodium ions neutralize the negative charges carried by the phosphate groups. They then form hydrogen bonds with the exposed bases G6 and G19 at the recognition sites, respectively, leading to a decrease in system free energy by 4.6 kcal mol^–1^ from step ① to step ② (Figure 10). In the transition from step ② to step ③, one of the AMP molecules approaches the aptamer at Site 2 (in the loop region), favorably positioning the OH group of the AMP sugar ring toward the phosphate oxygen atom of residue A10 of the aptamer backbone, facilitating further binding of the ligand. This process lowers the system free energy by 1.6 kcal mol^–1^. Moving from step ③ to step ④, the base portion of AMP at Site 1 forms an AMP:N6···N3:A23 hydrogen bond. The phosphate group of AMP at Site 1 thus displaces the hydrogen bond formed between AMP at Site 2 and base G19, resulting in a reduction in system free energy by 1.9 kcal mol^–1^. At this point, neither of the AMP molecules have yet formed G:AMP reverse Hoogsteen base pairs with bases G22 and G9. Steps ④-⑤-⑥ indicate that the AMP molecules at Sites 2 and 1 sequentially adjust their binding poses. The AMP in Site 2 approaches from the near-backbone side, and the AMP in Site 1 approaches in from the solvent side, consistent with free energy pathways in Figure 5. The two AMPs form hydrogen bonds with G9 and G22, respectively, and fully enter the binding pockets, resulting in a decrease in system energy by 11.4 kcal mol^–1^. The average binding free energy per ligand is −7.45 kcal mol^–1^, consistent with the measured binding free energy −6.5 kcal mol^–1^ through ITC experiments^17,18^ and −5.4 kcal mol^–1^ predicted by calculations.^26^ Thus, our enhanced sampling dynamics and free energy calculations provide a molecular-level explanation for the weak positive cooperative binding, suggesting that the main reason is the electrostatic interactions between the two AMP ligands through the phosphate groups and sodium ions. Mg^2+^ ions are often found in nucleic acid-containing experiments since it can more effectively bond to phosphate mainly by electrostatic forces. Herein, role of cations common in biological systems in the recognition mechanism is significantly highlighted through formation of the anion–cation–anion structural model. The present results also show that experimentally elevated salt concentration can be a key factor favoring the anion–cation–anion structural formation and thus promoting the recognition of the two sites to two AMP molecules.^52^

To compare the cooperative effects of recognition, we also constructed a suboptimal recognition pathway deviating from the minimum free energy pathway, represented by the solid pink curve in Figure 10. This pathway represents an independent recognition mechanism, where the AMP ligand fully enters the recognition Site 1 and stabilizes before Site 2 binds the second AMP molecule. The independent recognition pathway is higher by 2.1 kcal mol^–1^ than the weak cooperative binding case. Free energy calculations and dynamic structural analysis (Figure S17) suggest that if the ligands follow an independent binding mode, the stable hydrogen bonds formed by the phosphate group with G19 and G6 will increase the energy barrier required for adjusting the binding pose. Hence, connecting to a previous study, increasing the number of base pairs between the two recognition sites promotes rigid structure formation in chemical environments and reduces the intensity of the cooperative effect, with the Hill coefficient’n’ decreasing from 1.3 to 1.1^18^.

To further highlight the role of the phosphates in the AMP ligands, we also conducted 2 μs equilibrium dynamics simulations of the dual-site adenosine (ADN), rather than AMP, recognition model. The same biased sampling dynamics was carried out with the adenosines in both recognition sites, and the corresponding binding free energy landscapes were obtained and are shown in Figure S18. The results indicate that adenosine in Site 2 exhibits a smaller binding free energy, and clearly suggest an independent binding mechanism in the adenosine system, lacking the phosphate groups of the AMPs. Our studies hence support the weakly and positively cooperative mechanism of AMP binding and clearly show that the phosphate group of AMP plays an unexpected role in the cooperative recognition.

## Conclusions

We have systematically investigated the dual-site recognition mechanism of the widely used AMP-DNA nucleic acid aptamer using enhanced sampling molecular dynamics simulations. Through comparative studies between Site 1/AMP, Site 2/AMP and Sites 1,2/2AMP, we first discovered the impact on the affinities caused by an inequivalence in chemical environments between the two recognition sites in terms of spatial structure, binding free energy, and interaction decomposition, despite their identical base sequences. The additional hydrogen bond formed between the phosphate group of AMP and G6 near the 5′ to 3′ terminus endows the recognition site closer to the terminus (Site 1) slightly stronger binding free energy, measured as −6.88 ± 0.87 kcal mol^–1^ for Site 1 + AMP →Site 1/AMP, as compared to −4.25 ± 0.47 kcal mol^–1^ for Site 2/AMP formation, consistent with reported experimental values. Additional hydrogen bonds in Sites 1,2/2AMP contribute largely to the increasing stability of the dual binding model, and thus to the second AMP recognition. The binding free energies are increased to −8.53 ± 0.60 kcal mol^–1^ for the Site 2/AMP + AMP → Sites 1,2/2AMP and −9.48 ± 0.33 kcal mol^–1^ for the Site 1/AMP + AMP → Sites 1,2/2AMP processes.

Two-dimensional free energy landscapes and minimum free energy pathway calculations based on centroid distance and approaching angle as collective variables indicate two distinct recognition pathways with different energy barriers for the AMP ligands. The pathways of the dual-site recognition and other comparative studies demonstrate that the electrostatic interaction between the phosphate groups of the two AMP ligands and sodium cations contribute positively to the cooperative binding mechanism observed.

## Data Availability

Gromacs simulation data sets from the molecular dynamics trajectories of the Site 1/AMP, Site 2/AMP and Sites 1,2/2AMP simulations are available as download free of charge from zenodo.org; doi: 10.5281/zenodo.11395866.

## Supporting Information Available

Supplementary figures S1–S17 displaying RMSD curves from all replicas, PMF curves, free energy landscapes, and snapshots of recognition pathways (PDF)
