# Supplementary material for: Mechanism of Dual-Site Recognition in a Classic DNA Aptamer
Source: J Chem Inf Model. 2024 Sep 27;64(19):7698–708. doi: 10.1021/acs.jcim.4c01389 (PMC11481096; doi:10.1021/acs.jcim.4c01389)
Supplement: Supplementary file 1 — ci4c01389_si_001.pdf [file ci4c01389_si_001.pdf]

Supplementary Information

Mechanism of Dual-Site Recognition in a Classic DNA Aptamer

Yun-Peng Wang[1], Leif A. Eriksson [2,*] and Ru-bo Zhang[1,*]

[1]School of Chemistry and Chemical Engineering, Beijing Institute of Technology, South Street No. 5, Zhongguancun, Haidian District, 100081 Beijing, China and [2]Department of Chemistry and Molecular Biology, University of Gothenburg, Medicinaregatan 7b, 405 30 Goteborg, Sweden

FIGURES **PAGE**

S1. RMSD of the dual-site aptamer-AMP complex (Sites 1,2/2AMP)......3

S2. RMSD of the Site 1 aptamer-AMP complex (Site 1 /AMP)...............4

S3. RMSD of the Site 2 aptamer-AMP complex (Site 2 /AMP)...............5

S4. RMSF of the dual-site aptamer-AMP complex (Sites 1,2/2AMP). 6

S5. RMSF of the Site 1 aptamer-AMP complex (Site 1/AMP).................7

S6. RMSF of the Site 2 aptamer-AMP complex (Site 2/AMP).................8

S7. RMSD of the recognition sites in the dual-site aptamer-AMP complex (Sites 1,2/2AMP). 9

S8. RMSD of the recognition sites in the Site 1 aptamer-AMP complex (Site 2 /AMP)...10

S**S9.** RMSD of the recognition sites in the Site 2 aptamer-AMP complex (Site 2 /AMP)...11

S10. PMF curves of the Site 1 Recognition Model (Site 1 /AMP). 13

S11. PMF curves of the Site 2 Recognition Model (Site 2 /AMP). 13

S12. The global structure and the interaction between the two recognition sites in single recognition models..13

S13. Different pathways of AMP molecule recognition at Site 1 in the single-site recognition model..15

S14. The free energy landscape of Site 2 recognition to AMP molecule in the single-site recognition model...16

S15. Different pathways of AMP molecule recognition at Site 2 in the single-site recognition model..17

S16. Different pathways of AMP molecule recognition at Site 1 in the dual-site recognition model. 18
S17. Snapshots of the independent recognition process in the dual-site recognition model. 18
S18. Free energy landscape of the dual-site adenosine (ADN) recognition mechanism.. 18

TABLES **PAGE**

S1. RMSD Calculations for the Equilibrium Trajectories.......................12
S2. Basic molecular dynamics simulation information...........................20

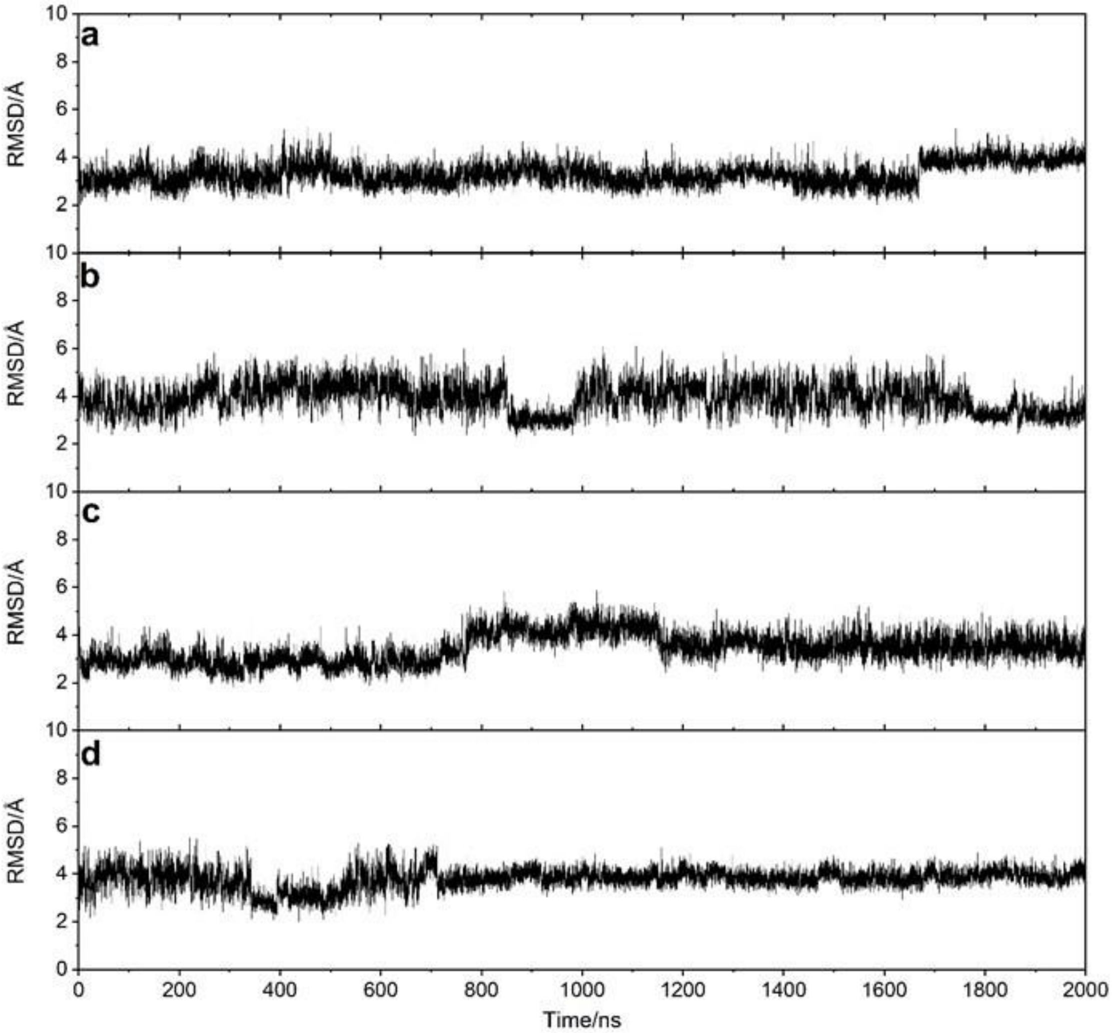

Figure S1. RMSDs from 2 μs MD simulations of the dual-site aptamer-AMP complex (Sites 1,2/2AMP). RMSD calculations are based on backbone heavy atoms. The trajectories labeled a, b, c, and d represent four parallel equilibrium simulation replicas. The primary cause of RMSD fluctuations around 1650 ns in replica a, around 900 ns and 1800 ns in replica b, and around 500 ns and 700 ns in replica d is the dissociation of Watson-Crick base pairs at the 5’ and 3’ ends.

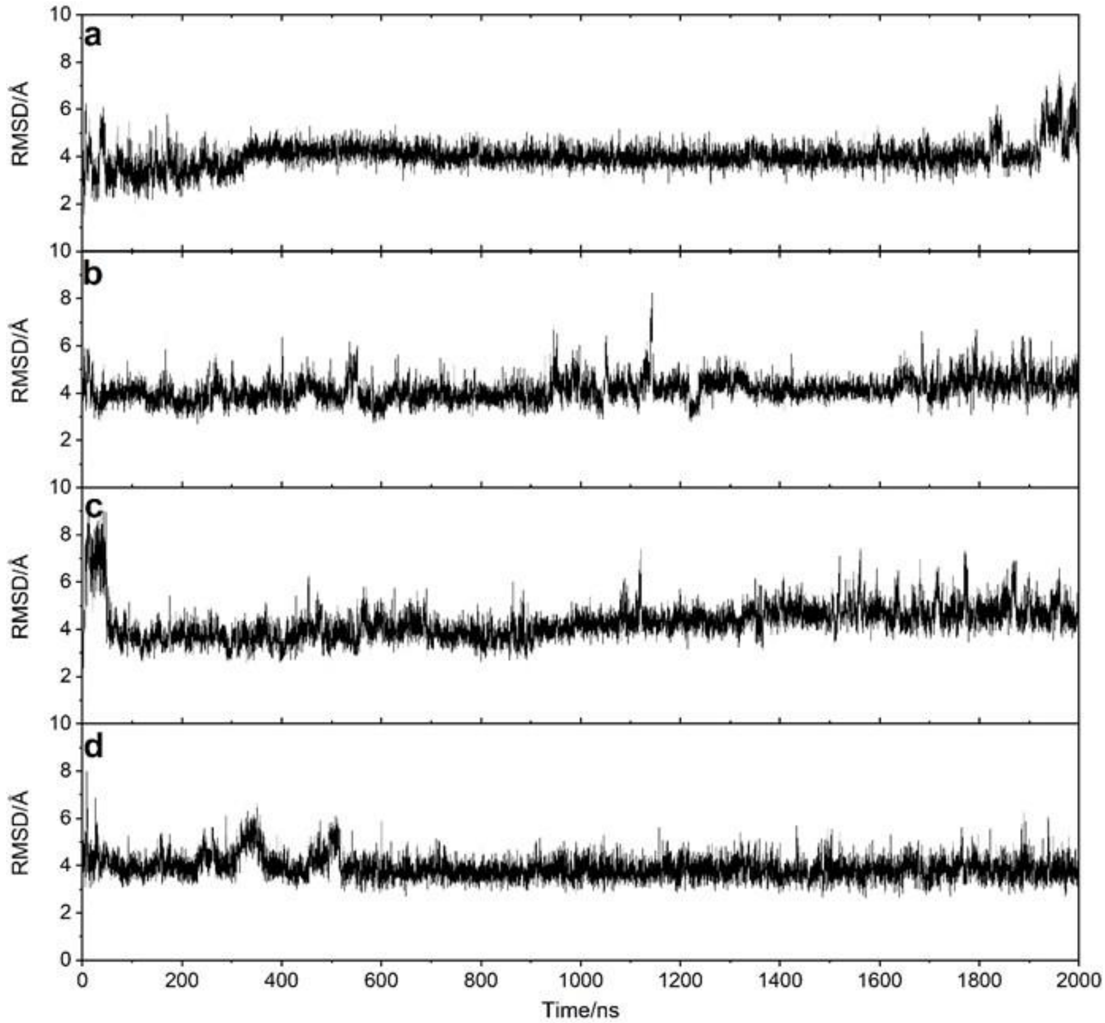

Figure S2. RMSDs from 2 μs MD simulations of the Site 1 aptamer-AMP complex (Site 1 /AMP). RMSD calculations are based on the backbone heavy atoms. The trajectories labeled a, b, c, and d represent four parallel equilibrium simulation replicas.

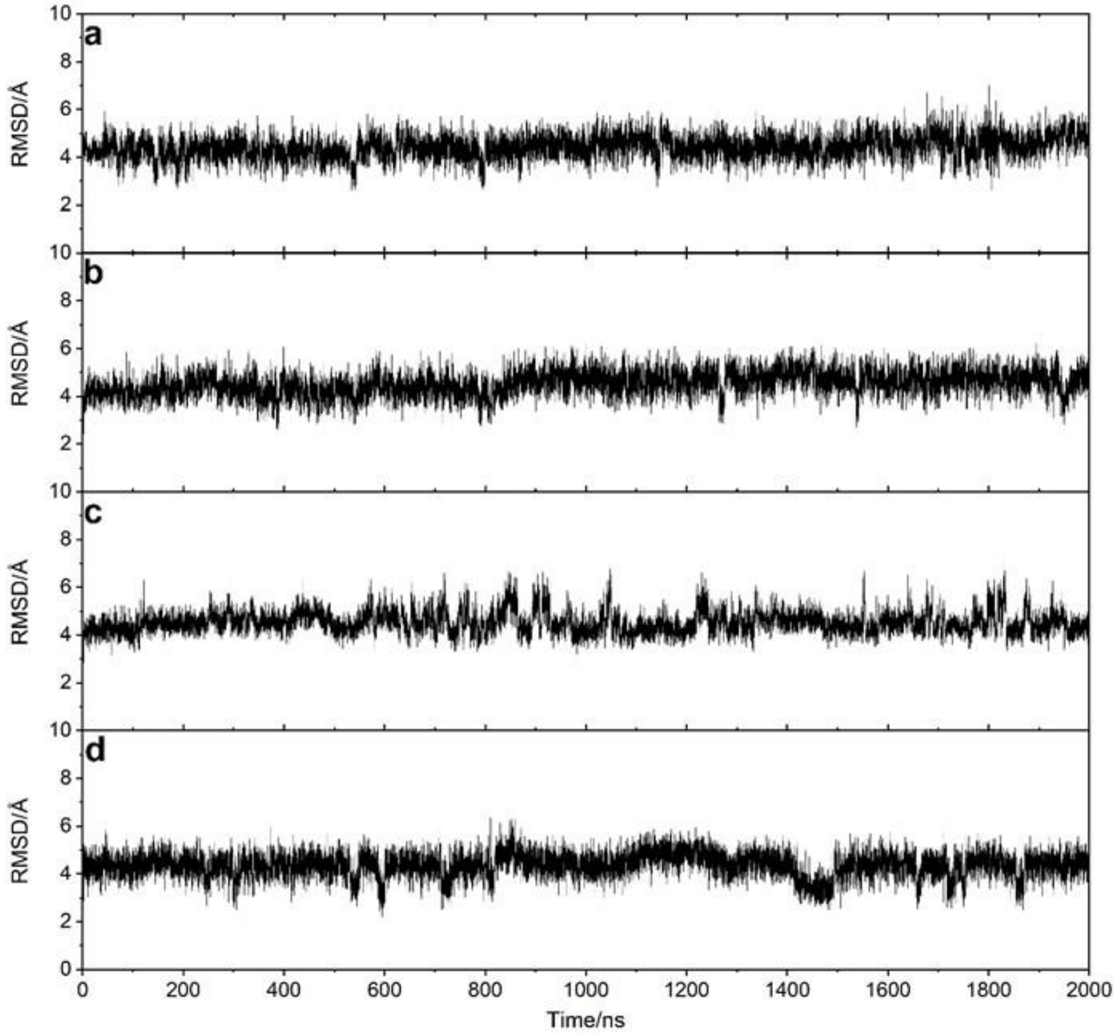

Figure S3. RMSDs from 2 μs MD simulations of the Site 2 aptamer-AMP complex (Site 2 /AMP). RMSD calculations are based on the backbone heavy atoms. The trajectories labeled a, b, c, and d represent four parallel equilibrium simulation replicas.

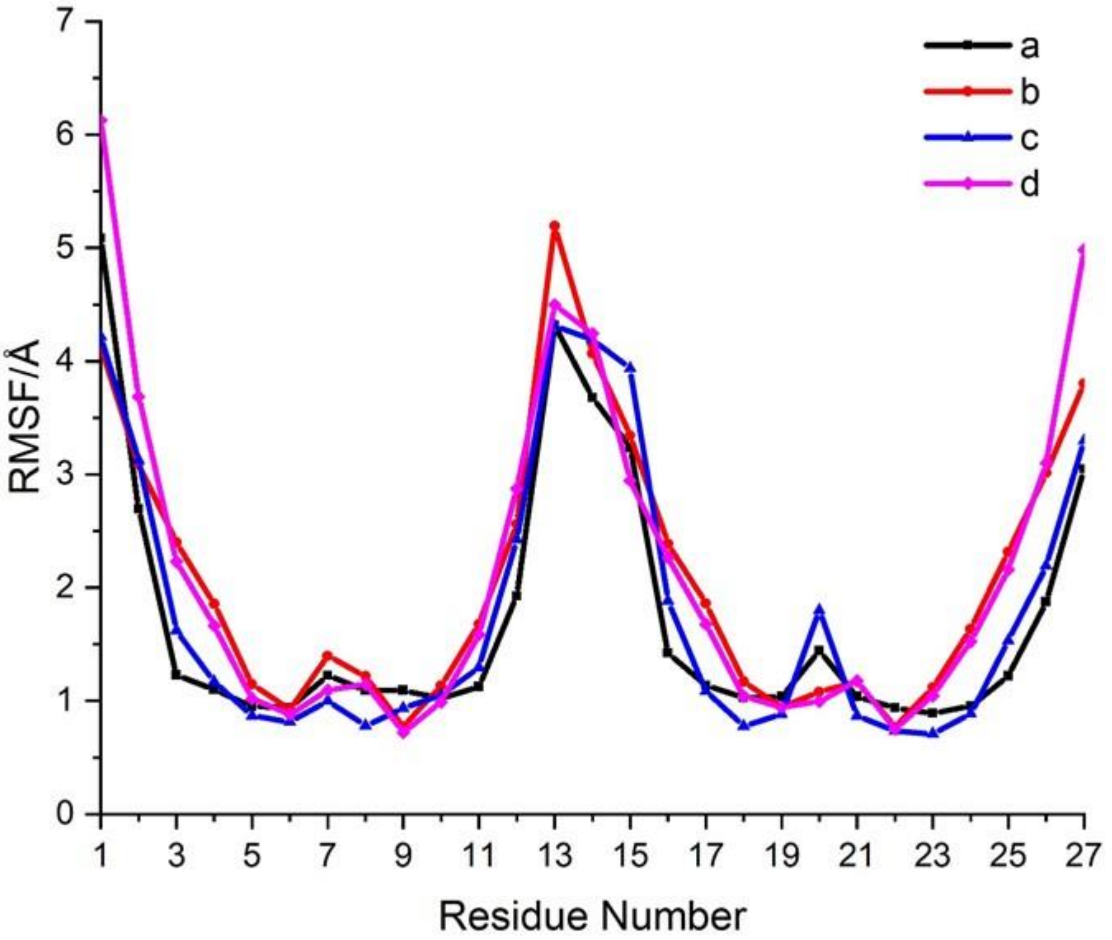

Figure S4. RMSF of the dual-site aptamer-AMP complex (Sites 1,2/2AMP). The curves labeled a, b, c, and d represent the four parallel 2 μs equilibrium simulation replicas (Figure S1). Residues with significant RMSF changes include A1, C2, G26, T27 (dissociation of 5’ and 3’ end base pairs); G7, A20 (flexibility of unpaired internal bulge residues); and T13, T14, T15 (movement of the loop region).

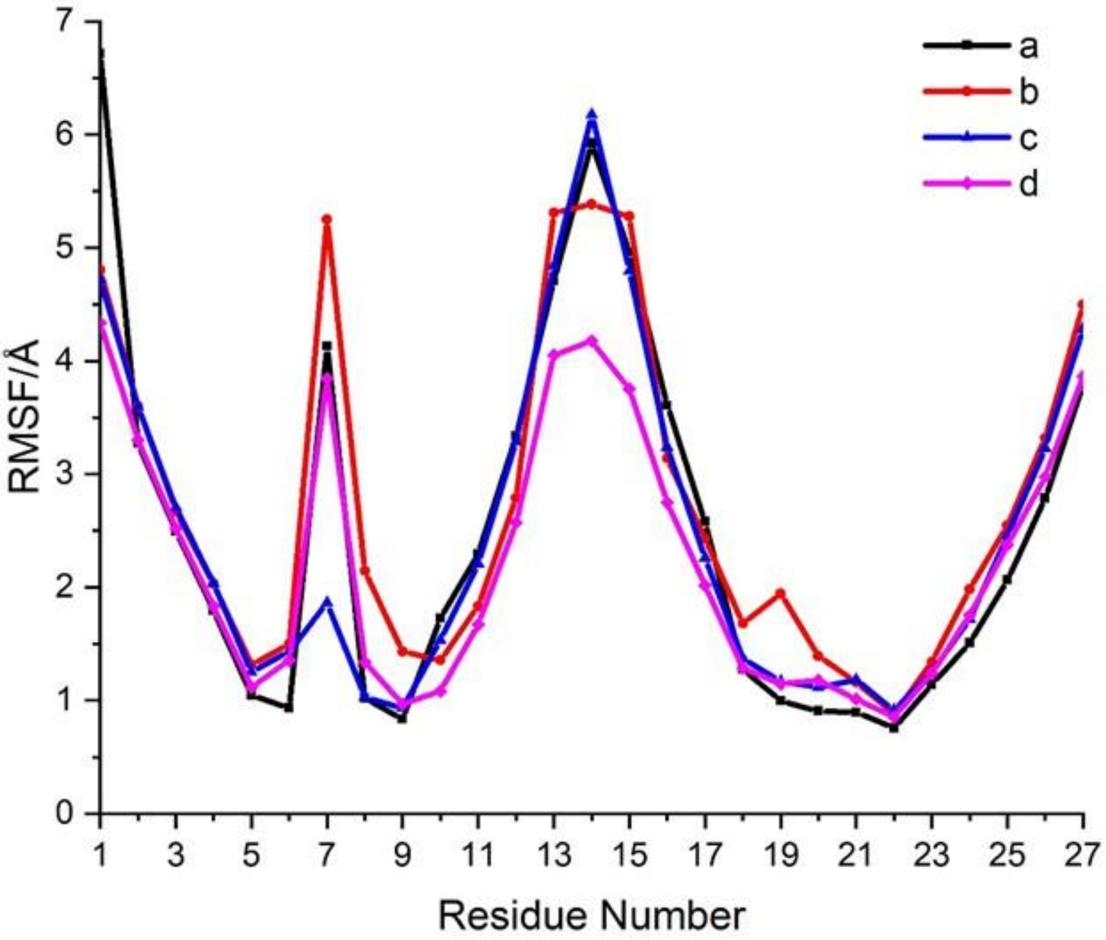

Figure S5. RMSF of the Site 1 aptamer-AMP complex (Site 1/AMP). The curves labeled a, b, c, and d represent the four 2 μs equilibrium simulation replicas (Figure S2). Residues with significant RMSF changes include A1, C2, G26, T27 (dissociation of 5' and 3' end base pairs); G7, A20 (flexibility of unpaired internal bulge residues); and T13, T14, T15 (movement of the loop region).

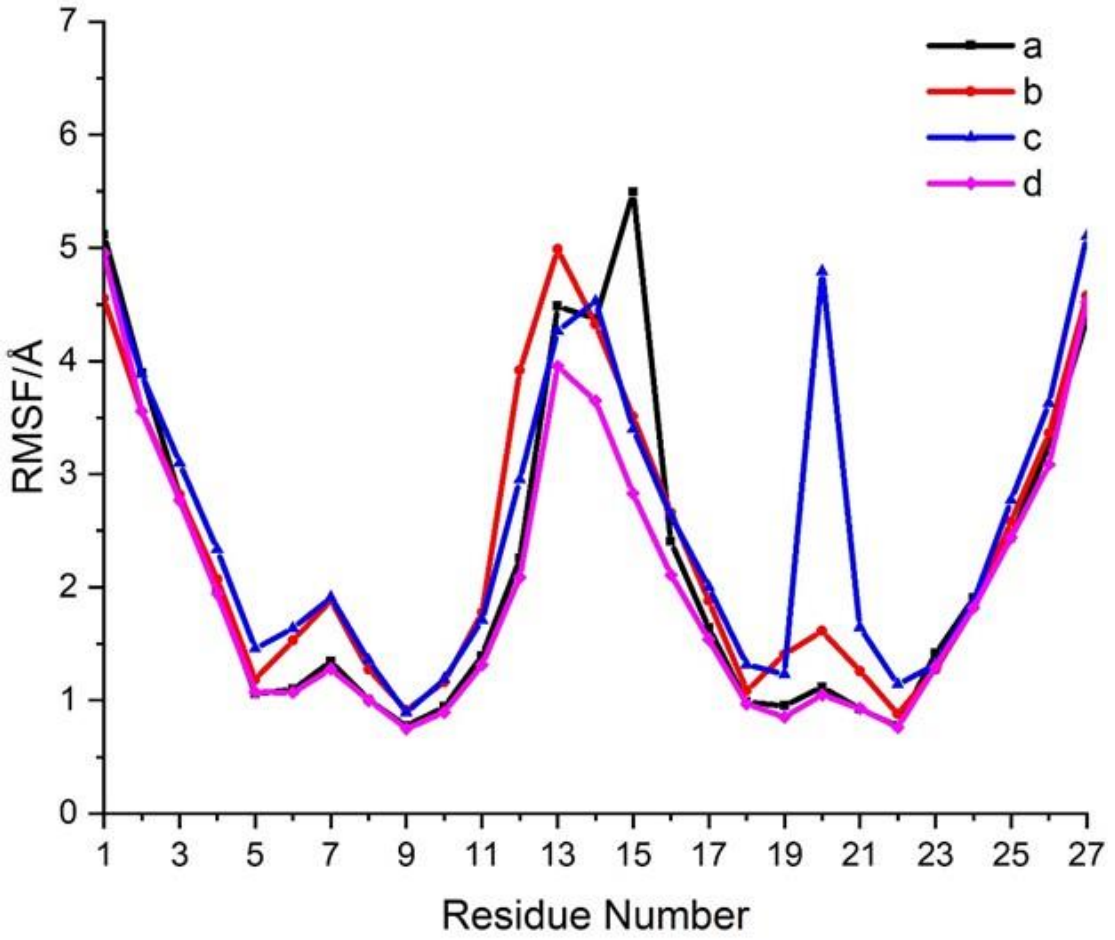

Figure S6. RMSF of the Site 2 aptamer-AMP complex (Site 2/AMP). The curves labeled a, b, c, and d represent the four 2 μs equilibrium simulation replicas (Figure S3). Residues with significant RMSF changes include A1, C2, G26, T27 (dissociation of 5' and 3' end base pairs); G7, A20 (flexibility of unpaired internal bulge residues); and T13, T14, T15 (movement of the loop region).

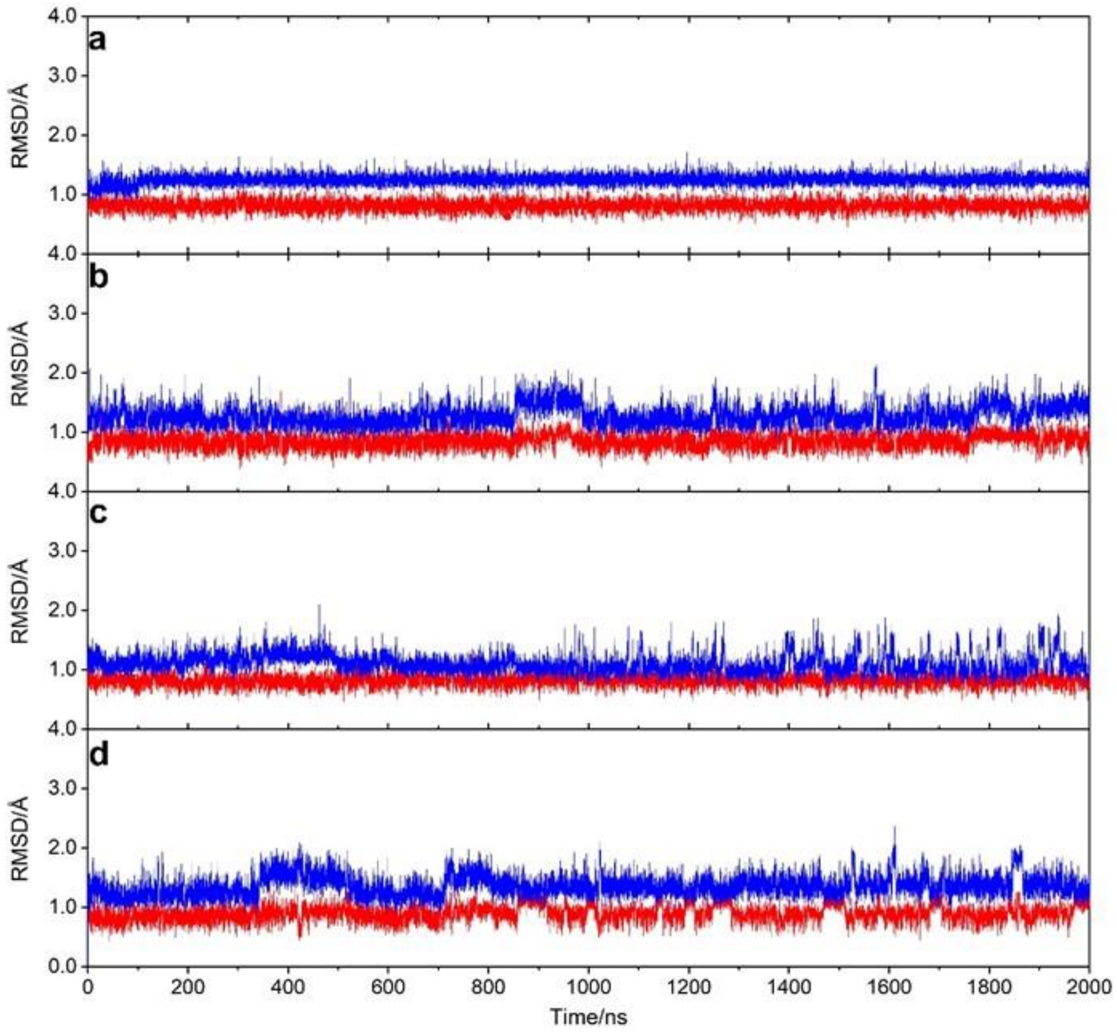

Figure S7. RMSD of the recognition sites in the dual-site aptamer-AMP complex (Sites 1,2/2AMP). Site 1 is represented by red curves, including residues G5, G6, G21, G22, A23. Site 2 is represented by blue curves, including residues G8, G9, A19, G18, G19. The labels a, b, c, and d denote the four parallel simulation trajectories (*cf.* Figure S1). The RMSD calculations are based on the heavy atoms of the recognition site residues.

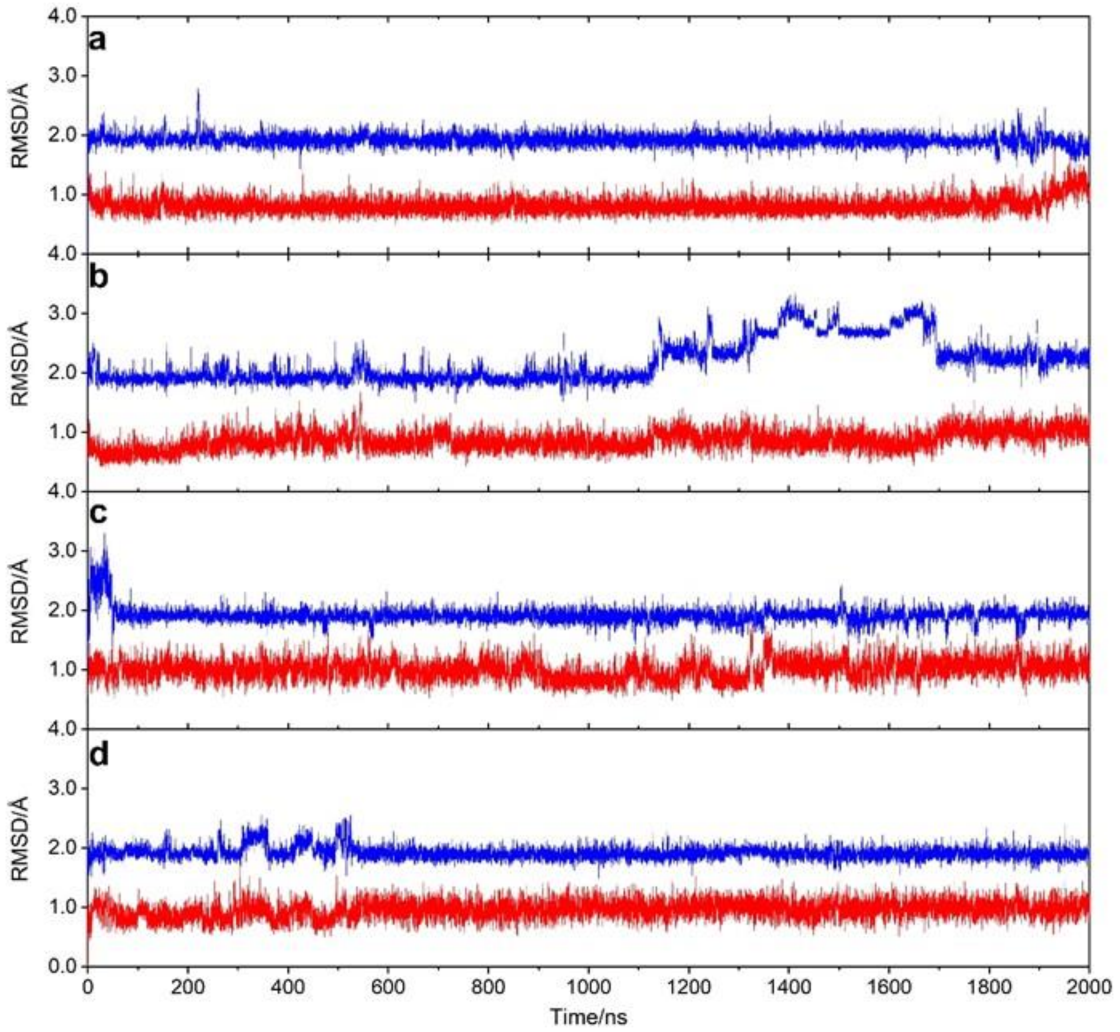

Figure S8. RMSD of the recognition sites in the Site 1 aptamer-AMP complex (Site 1 /AMP). Site 1 is represented by red curves, including residues G5, G6, G21, G22, A23. Site 2 is represented by blue curves, including residues G8, G9, A19, G18, G19. The labels a, b, c, and d denote the four parallel simulation trajectories (*cf.* Figure S2). The RMSD calculations are based on the heavy atoms of the recognition site residues. The fluctuations of Site 2 in Site 1/AMP replica b from 1100 ns to 1700 ns are due to the formation of a hydrogen bond between A20N7 and G8N2, which alters the conformation of Site 2 that does not bind AMP.

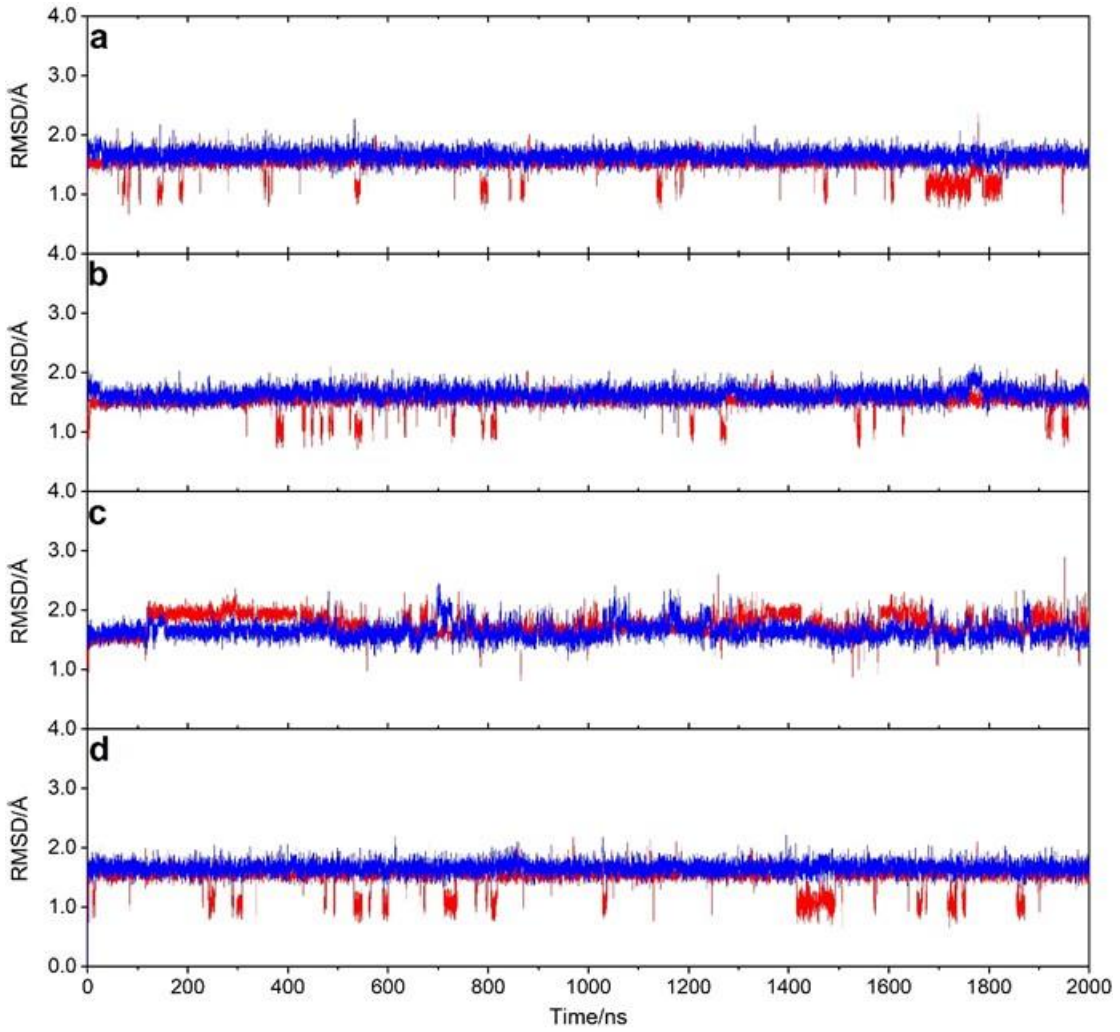

Figure S9. RMSD of the recognition sites in the Site 2 aptamer-AMP complex (Site 2 /AMP). Site 1 is represented by red curves, including residues G5, G6, G21, G22, A23. Site 2 is represented by blue curves, including residues G8, G9, A19, G18, G19. The labels a, b, c, and d denote the four parallel simulation trajectories (*cf.* Figure S3). The RMSD calculations are based on the heavy atoms of the recognition site residues.

Table S1. RMSD Values (average±SD, in Å) for the Equilibrium Trajectories. For each system, four 2μs MD simulations (a, b, c, d) were performed; *cf.* Figures S1 -S9.

System	Backbone	Site 1	Site 2
Site 1,2/2AMP-a	3.30±0.38	0.81±0.08	1.25±0.07
Site 1,2/2AMP-b	3.91±0.53	0.86±0.10	1.28±0.14
Site 1,2/2AMP-c	3.45±0.51	0.82±0.08	1.11±0.13
Site 1,2/2AMP-d	3.78±0.33	0.90±0.11	1.35±0.14
Site 1/AMP-a	4.01±0.37	0.82±0.10	1.91±0.08
Site 1/AMP-b	4.15±0.39	0.86±0.14	2.19±0.30
Site 1/AMP-c	4.31±0.56	1.01±0.14	1.92±0.09
Site 1/AMP-d	3.91±0.37	0.96±0.12	1.92±0.08
Site 2/AMP-a	4.41±0.39	1.51±0.11	1.65±0.08
Site 2/AMP-b	4.51±0.42	1.52±0.09	1.63±0.09
Site 2/AMP-c	4.53±0.37	1.75±0.14	1.64±0.11
Site 2/AMP-d	4.35±0.41	1.49±0.13	1.66±0.08

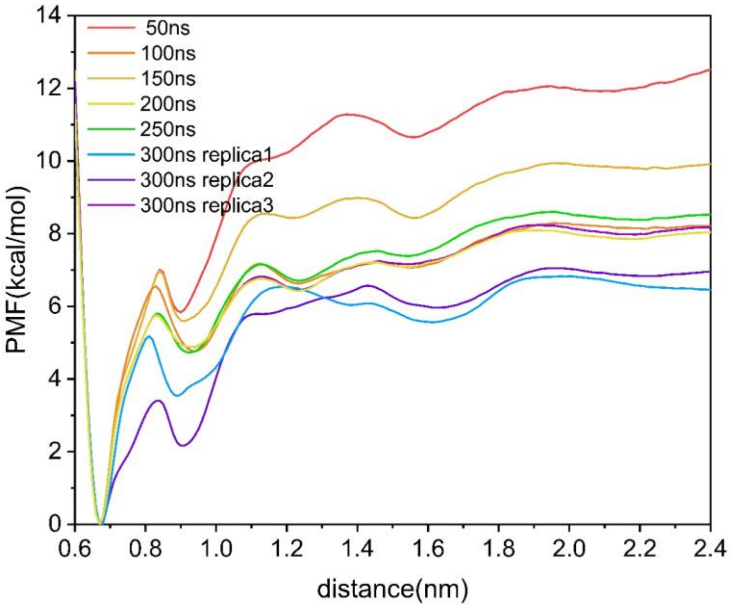

Figure S10. Potential of Mean Force (PMF) curves of the Site 1 Recognition Model (Site 1 /AMP). The different colors represent the PMF at different simulation times to assess the convergence of the WTM-eABF enhanced sampling.

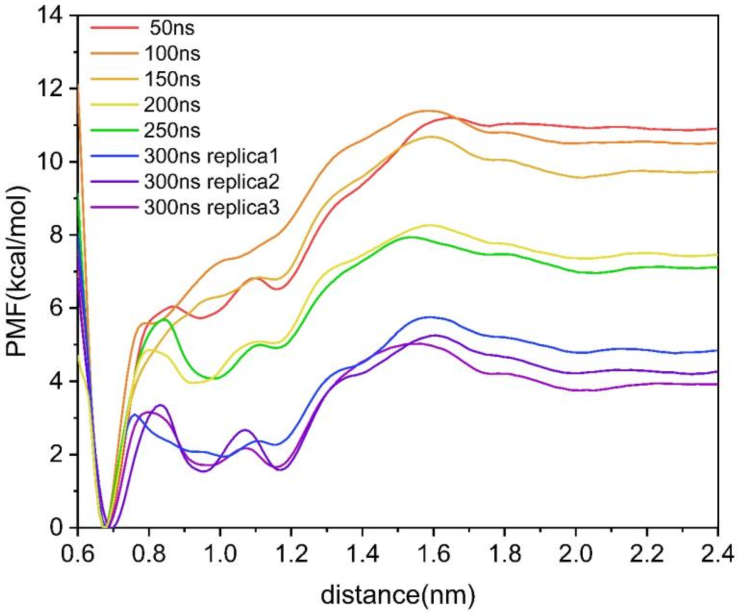

Figure S11. PMF curves of the Site 2 Recognition Model (Site 2 /AMP). The different colors represent the PMF at different simulation times to assess the convergence of WTM-eABF enhanced sampling.

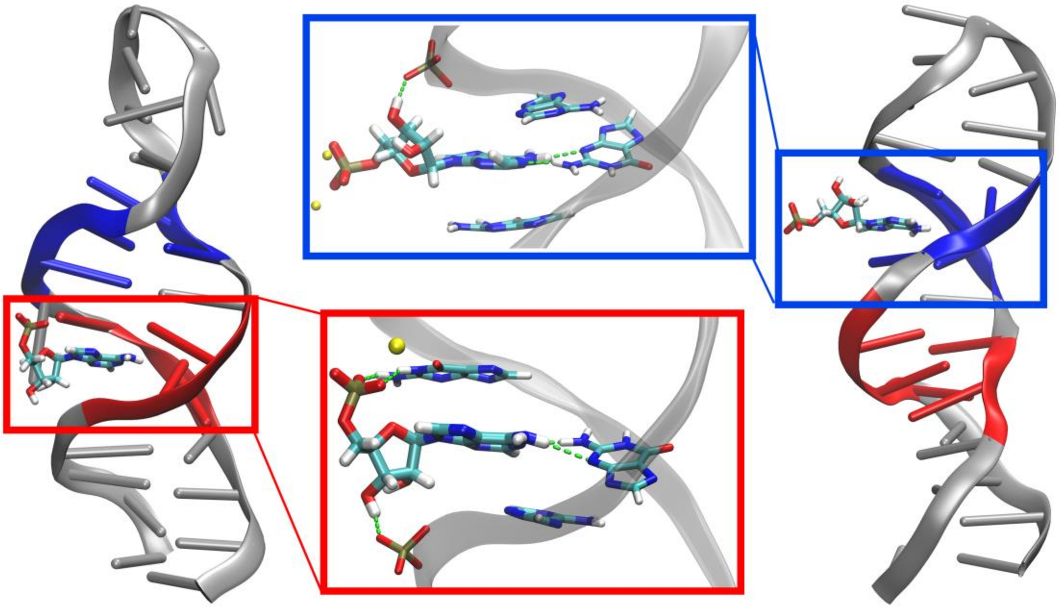

Figure S12. The global structure and the interaction between the two recognition sites in single recognition models. The red color indicates Site 1, which is near the 5'3' end, while the blue color represents Site 2, located near the LOOP region.

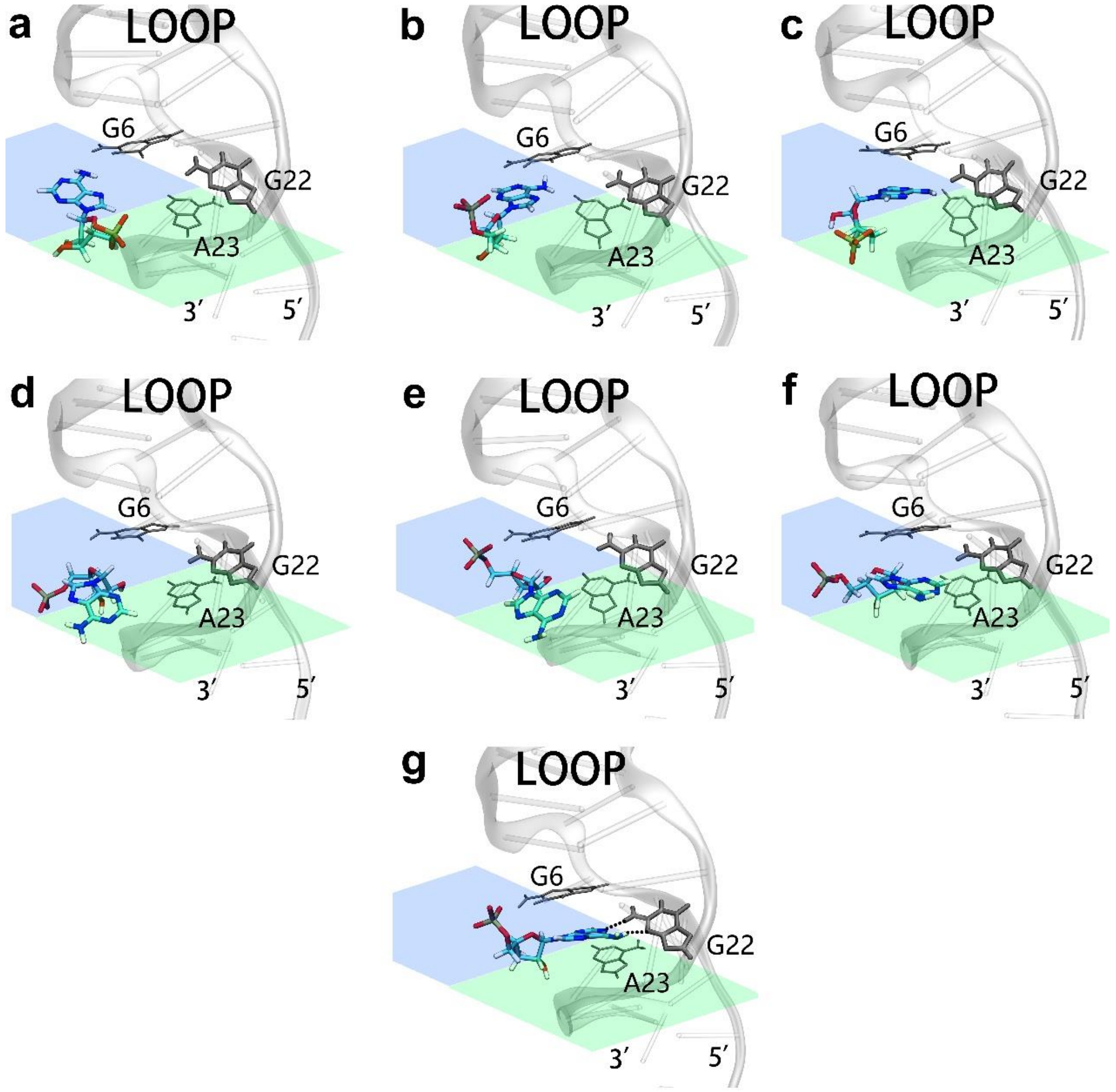

Figure S13. Different pathways of AMP molecule recognition at Site 1 in the single-site recognition model. The upper panels (a, b, c) represent the pathway of ligand binding from the solvent side (blue area), φ ranging from 0 to 180°, which corresponds to the minimum free energy pathway. The middle panels (d, e, f) represent the pathway of ligand binding from the near-backbone side (green area), φ ranging from -180° to 0, and corresponds to the suboptimal free energy pathway. The panel (g) at the bottom represents the final stable binding state.

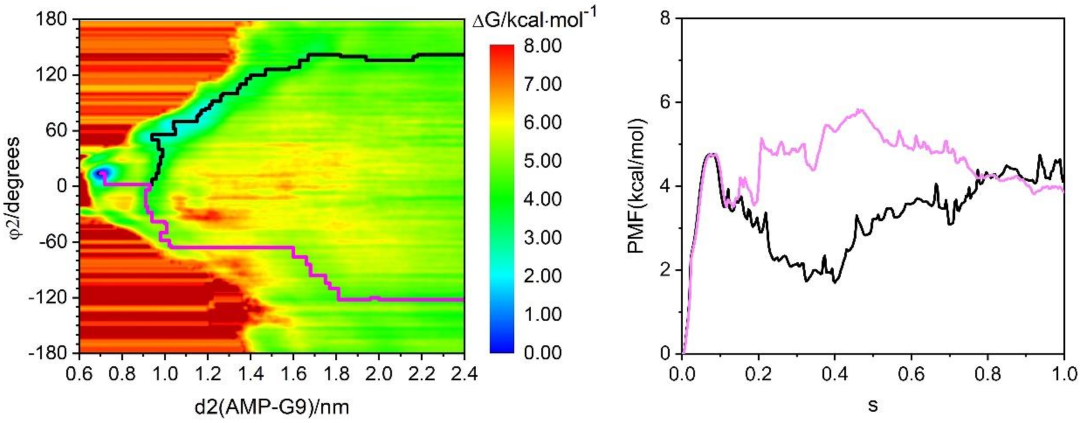

Figure S14. (left) Free energy landscape of Site 2 recognition of AMP in the single-site recognition model. The black curve is the minimum free energy pathway and represents the optimal recognition pathway. The pink curve is the suboptimal recognition pathway relative to the theoretical black curve. (right) Potential of Mean Force (PMF) curves along the minimum free energy pathway and the suboptimal recognition pathway. The horizontal axis represents the variable 's' indicating the progress along the collective variables of the respective paths.

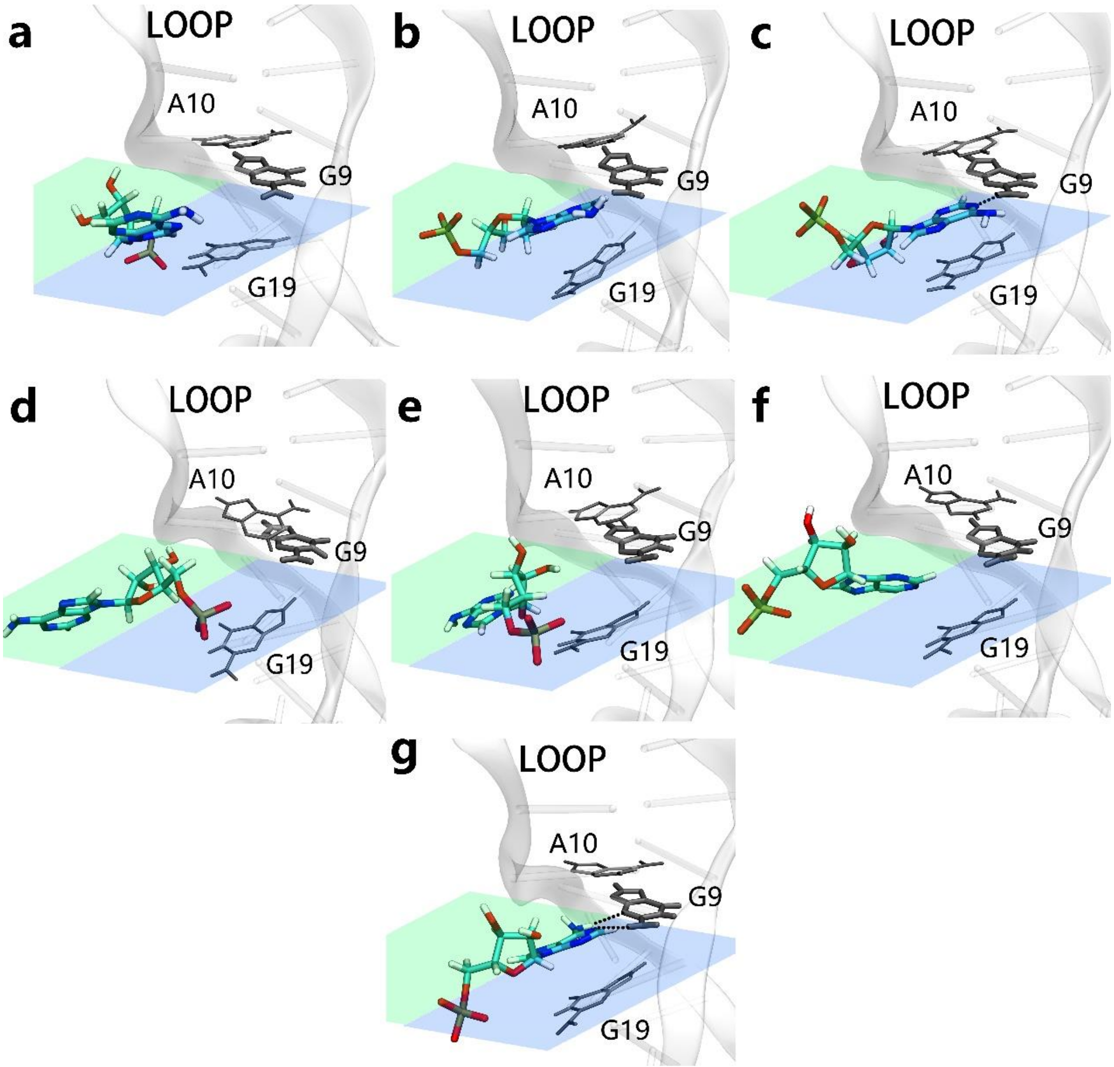

Figure S15. Different pathways of AMP molecule recognition at Site 2 in the single-site recognition model. The upper panels (a, b, c) represent the pathway of ligand binding from the solvent side (blue region), φ ranging from 0 to 180°, which corresponds to the minimum free energy pathway. The middle panels (d, e, f) represent the pathway of ligand binding from the near-backbone side (green region), φ ranging from -180° to 0, and corresponds to the suboptimal free energy pathway. The panel (g) at the bottom represents the final stable binding state.

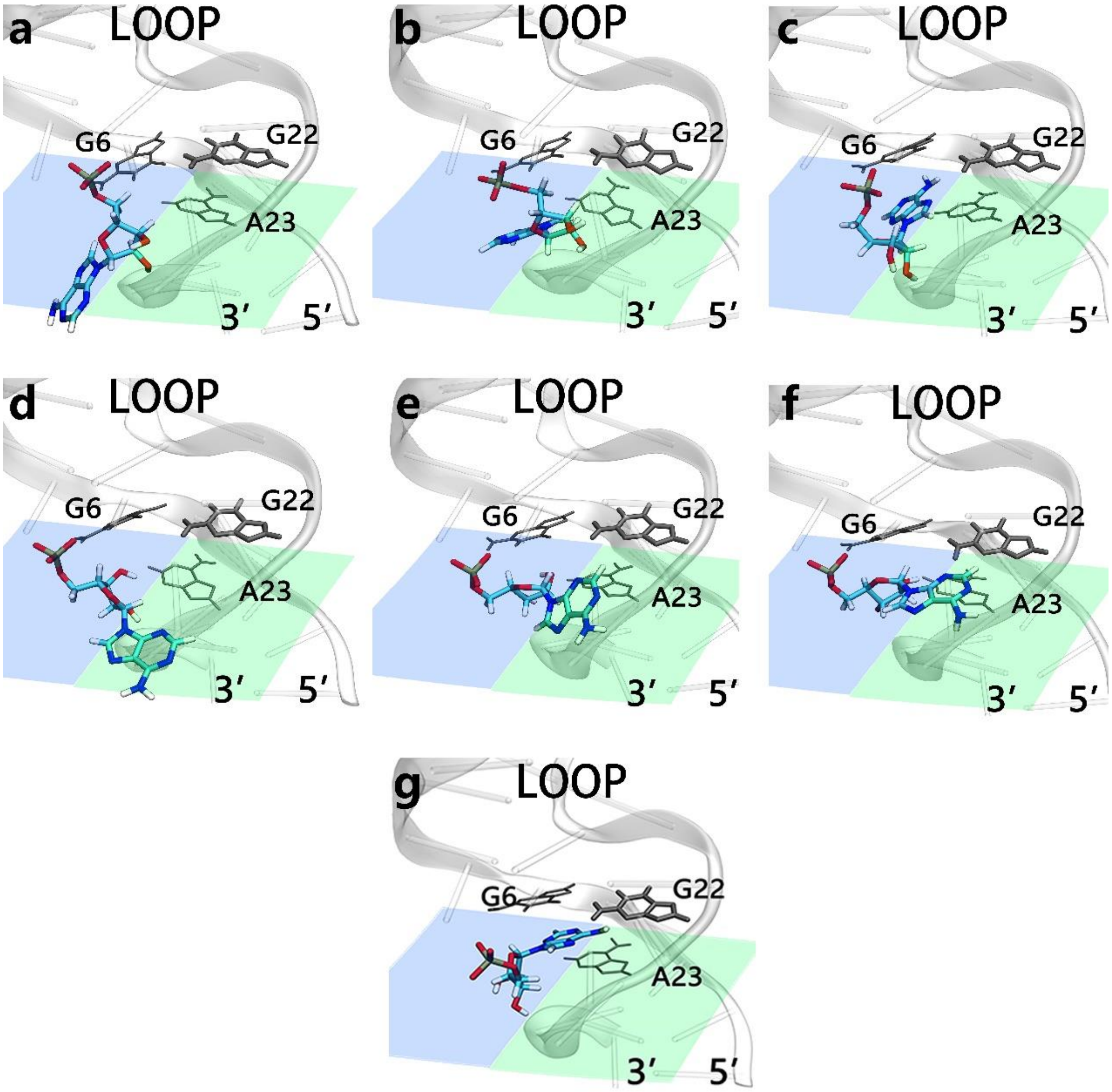

Figure S16. Different pathways of AMP molecule recognition at Site 1 in the dual-site recognition model. The upper panels (a, b, c) represent the pathway of ligand binding from the solvent side (blue region), φ ranging from 0 to 180°, which corresponds to the minimum free energy pathway. The middle panels (d, e, f) represent the pathway of ligand binding from the near-backbone side (green region), φ ranging from -180° to 0, and corresponds to the suboptimal free energy pathway. The panel (g) at the bottom represents the final stable binding state.

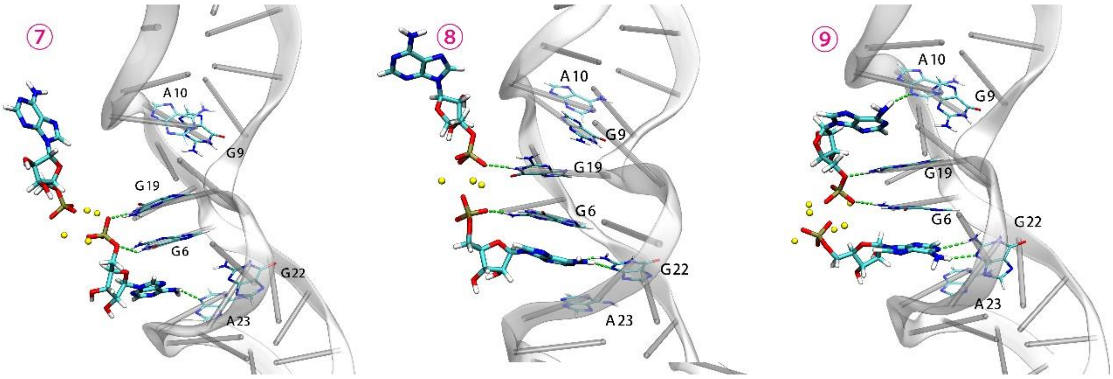

Figure S17. Snapshots of the independent recognition process in the dual-site recognition model. The snapshots are arranged according to the order of ligand association corresponding to steps ⑦, ⑧ and ⑨ along the suboptimal binding pathway, respectively, as indicated in Figure 10b of the main text.

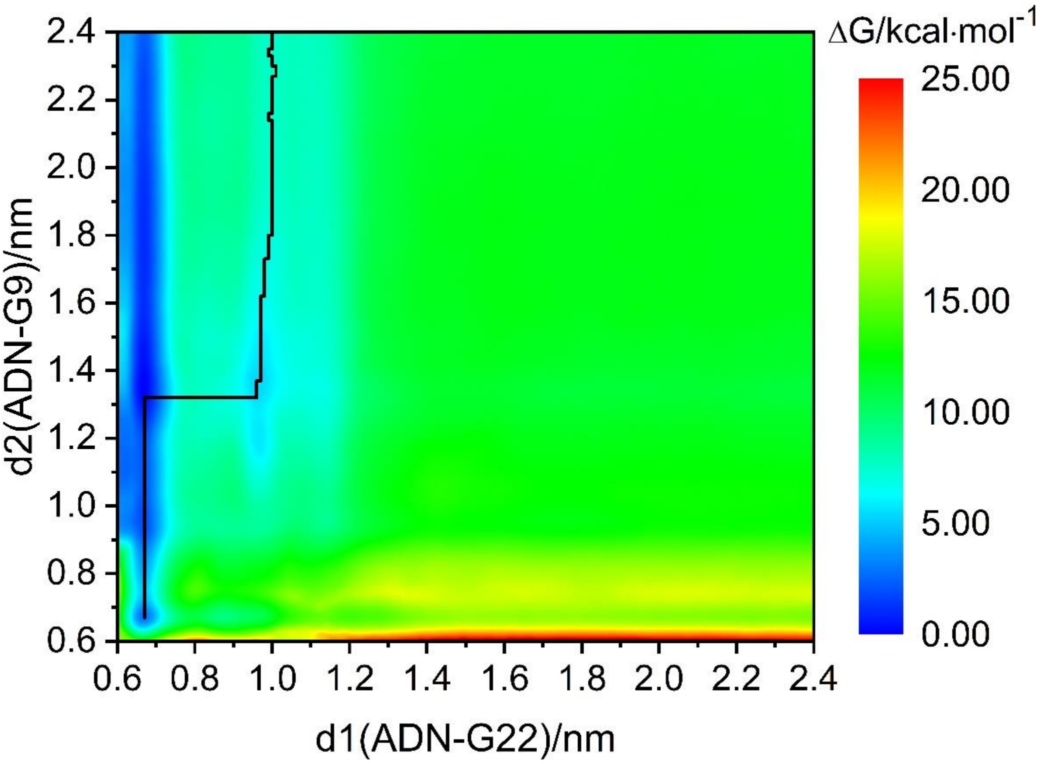

Figure S18. Free energy landscape of the dual-site adenosine (ADN) recognition mechanism. The horizontal axis represents the distance between AND_1 and the centroid of base G22 in Site 1. The vertical axis represents the distance between AND_2 and the centroid of base G9 in Site 2. The black curve represents the minimum free energy pathway.

Table S2. Basic molecular dynamics simulation information in this research.

Type	CV	System	Biased AMP	length	replica
Conventional	None	Site 1/AMP	None	2000ns	4
Conventional	None	Site 2/AMP	None	2000ns	4
Conventional	None	Site 1,2/2AMP	None	2000ns	4
1D PMF	Distance	Site 1/AMP	Site 1	300ns	3
1D PMF	Distance	Site 2/AMP	Site 2	300ns	3
2D PMF	Distance<br>Approaching angle	Site 1/AMP	Site 1	1200ns	1
2D PMF	Distance<br>Approaching angle	Site 2/AMP	Site 2	1200ns	1
2D PMF	Distance<br>Approaching angle	Site 1,2/2AMP	Site 1	1200ns	1
2D PMF	Distance<br>Approaching angle	Site 1,2/2AMP	Site 2	1200ns	1
2D PMF	Distance<br>Distance	Site 1,2/2AMP	Both	1200ns	1
